# *Viridibacillus* culture derived silver nanoparticles exert potent anticancer action in 2D and 3D models of lung cancer via mitochondrial depolarization-mediated apoptosis

**DOI:** 10.1016/j.mtbio.2024.100997

**Published:** 2024-02-11

**Authors:** Abhayraj S. Joshi, Mugdha V. Bapat, Priyanka Singh, Ivan Mijakovic

**Affiliations:** a The Novo Nordisk Foundation Center for Biosustainability, Technical University of Denmark, Kongens Lyngby, Denmark; b Department of Biology and Biological Engineering, Division of Systems and Synthetic Biology, Chalmers University of Technology, Sweden

**Keywords:** Anticancer silver nanoparticles, Non-small cell lung cancer, A549 spheroids, 3D cell culture, Mitochondrial depolarization, Intrinsic apoptosis pathway

## Abstract

Lung cancer is one of the most commonly occurring cancer types that accounts for almost 2 million cases per year. Its resistance to anticancer drugs, failure of new molecules in clinical trials, severe side-effects of current treatments, and its recurrence limit the success of anticancer therapies. Nanotherapeutic agents offer several advantages over conventional anticancer therapies, including improved retention in tumors, specificity, and anticancer effects at lower concentrations, hence reducing the side-effects. Here, we have explored the anticancer activity of silver nanoparticles synthesized in *Viridibacillus* sp. enriched culture medium for the first time. Such green nanoparticles, synthesized by biological systems, are superior to chemically synthesized ones in terms of their environmental footprint and production cost, and have one crucial advantage of excellent stability owing to their biological corona. To assess anticancer activity of these nanoparticles, we used conventional 2D cultured A549 cells as well as 3D spheroids of A549 cells. In both models of lung cancer, our silver nanoparticles diminished cell proliferation, arrested DNA synthesis, and showed a dose dependent cytotoxic effect. The nanoparticles damaged the DNA and mitochondrial structures in both A549 cells and A549 spheroids, leading to mitochondrial depolarization and increased cell permeability. Low lethal median doses (LD50) for 2D cultured A549 cells (1 μg/ml) and for A549 spheroids (13 μg/ml) suggest that our nanoparticles are potent anticancer agents. We also developed *in vitro* tumor progression model and *in vitro* tumor size model using 3D spheroids to test anticancer potential of our nanoparticles which otherwise would require longer experimental duration along with large number of animals and trained personnel. In these models, our nanoparticles showed strong dose dependent anticancer activity. In case of *in vitro* tumor progression model, the A549 cells failed to form tight spheroidal mass and showed increased dead cell fraction since day 1 as compared to control. On the other hand, in case of *in vitro* tumor size model, the 4 and 8 μg/ml nanoparticle treatment led to reduction in spheroid size from 615 ± 53 μm to 440 ± 45 μm and 612 ± 44 μm to 368 ± 62 μm respectively, within the time span of 3 days post treatment. We believe that use of such novel experimental models offers excellent and fast alternative to *in vivo* studies, and to the best of our knowledge, this is the first report that gives proof-of-concept for use of such novel *in vitro* cancer models to test anticancer agents such as *Viridibacilli* culture derived silver nanoparticles. Based on our results, we propose that these nanoparticles offer an interesting alternative for anticancer therapies, especially if they can be combined with classical anticancer drugs.

## Graphical abstract

**Figure:**
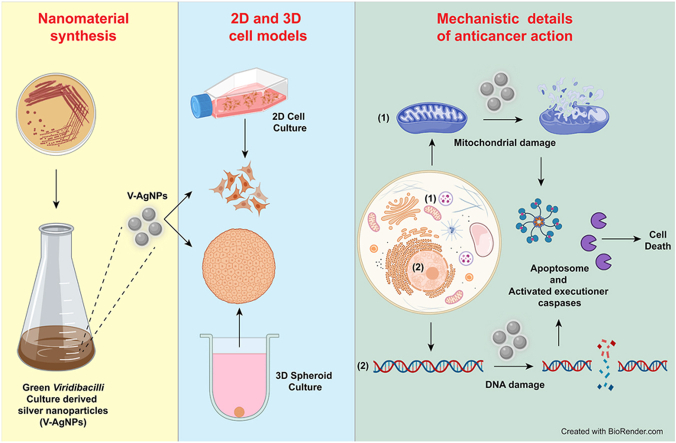

## 1 Introduction

Great advances have been achieved in the synthesis and characterization of nanomaterials of various origins [1,2]. Nanomaterials within the size range of 1–100 nm are employed in pharmaceutical, cosmeceutical, agricultural, biotechnological, electronics, and electrical industries owing to their unique physical, chemical, optical, and electrical properties [1,2]. Particularly in the pharmaceutical field, the nanomaterials have revolutionized the therapeutic and diagnostic potential of existing drugs, novel therapeutic molecules, and biosensors by several folds. Specifically in the field of cancer, nanomaterials have provided outstanding advantages owing to their small size [1,2].

Cancer is the second leading cause of death across the globe. As a life-threatening non-communicable disease, cancer severely affects the quality of patient's life and puts a severe burden on healthcare systems [3,4]. Among all types of cancers, the lung cancer is the most frequently diagnosed one (∼2 million new cases/year) and accounts for almost 20% of all cancer deaths [5,6]. Research driven towards understanding the cancer biology has led to the discovery of novel targets and novel therapies for treatment of cancer [3,4]. However, irrespective of advances in both cancer biology and cancer therapy fields, no definitive treatment is currently available. Current cancer therapy is divided into two subcategories: the conventional means such as chemotherapy and/or radiotherapy and the novel means, such as immunotherapy and gene therapy [[3], [4], [5]]. However, both approaches suffer from disadvantages such as severe side-effects, resistance to the treatment by tumor cells owing to prolonged therapy and/or genetic mutation, limited success in case of metastatic cancers, and cancer recurrence. Moreover, gene therapy and immunotherapy are still in their infancy. Therefore in such scenario, the nanomaterials pose as a better alternative [7].

Various types of nanomaterials (polymeric, inorganic, metallic, lipidic, proteinaceous) have been explored for analyzing their anticancer potentials [7]. Few of them such as paclitaxel loaded albumin nanoparticles (Abraxane®) have been already approved by Food and Drug Administration (FDA, USA) for treating breast cancer, non-small cell lung cancer, and pancreatic cancer [8,9]. The small size of nanoparticles offers enhanced permeation and retention (EPR) effect in tumor. Since the surface of nanoparticles is amenable for modification, targeted delivery to tumor cells and/or particular cell organelle is possible. Specific targeting via nanomaterials allows improvement of therapeutic indices of the drugs and thereby reduces their side-effects. The nanomaterials provide protection to sensitive drugs in tumor microenvironment that possesses low pH, hypoxia, and high proteases contents. Nanomaterials are also capable of co-delivery of multiple anticancer drugs and their controlled release at tumor site [7,10]. Therefore, the field of anticancer nanomaterials has grown exponentially in the past decade [7]. Among these types of nanomaterials, the silver nanomaterials; also termed as nano-silver or silver nanoparticles (AgNPs) have received quite an attention in recent years. Since ancient times, the use of silver solutions and silver salts has been prescribed for medical purposes [11]. And till date, according to the ‘Nanotechnology Product Database’, there are approximately >1200 silver nanomaterial products that have been employed for their applications in biomedical, cosmetic, agricultural, food, packaging, textile, and marine industries [12]. In recent years, AgNPs have shown remarkable anticancer properties against various types of cancers [13]. AgNPs can be synthesized using various physical, chemical, and biological methods [[14], [15], [16], [17]]. The physical methods employ special source of energy, like ultrasound or microwaves. In these methods, the physical energy (e.g.: ultrasound, microwaves, laser irradiation, or γ-irradiation) either leads to thermal decomposition of the silver or creates hydrated electrons as well as radicals via microwaves/γ-irradiation in the aqueous solution of silver salts and reduce silver ions to yield AgNPs. Owing to high energy of physical methods, AgNPs can be synthesized in short amount of time [[14], [15], [16]]. On the other hand, the chemical methods involve a range of organic/inorganic chemicals which reduce silver ions to elementary state and thereby form nanoparticles. But both, physical methods and chemical methods suffer with serious drawbacks such as use of high energy source, requirement of special equipment and trained personnel, and use of hazardous chemicals [[14], [15], [16]]. Alternative to these methods, the biological methods, have gained much popularity in recent years. These biological methods employ bacteria, fungi, or plants to synthesize AgNPs of varying sizes and shapes and they are also known as “green synthesis” methods due to their environmental friendly nature [17]. Production of large amount of AgNPs from simple resources make them cost-effective and economically viable. The biological components present in growth media or extracts (carbohydrates, proteins etc.) help in reducing the silver salts to form AgNPs. These components also adsorb on the surface of AgNPs to form a biological corona. Owing to this corona, these AgNPs show excellent stability over longer periods of times [[14], [15], [16], [17]]. Several reports show that such “green AgNPs” have antibacterial, anti-inflammatory, and anticancer activities [18]. It has been shown that the AgNPs show antibacterial activity by interacting with bacterial cell membrane, bacterial proteins, and bacterial nucleic acids; whereas, they show anti-inflammatory properties by reducing the expression of pro-inflammatory cytokines such as interleukin-6, interleukin-1β [18]. According to the extensive review published by Mariana Morais et al. the green AgNPs have been proven to be active against breast cancer, lung cancer, head and neck cancer, gynecologic cancer, urologic cancer, digestive system cancer, skin cancer, brain cancer, blood cancer, and brain cancer [19]. The mechanism of anticancer action of AgNPs obtained from various green sources differs among different types of cancers in terms of cellular changes ultimately leading to cell death [19].

In presented research, we have reported anticancer activity of the “green” AgNPs synthesized from *Viridibacilli* enriched culture medium (henceforth termed as V-AgNPs) against 2D and 3D models of a non-small cell lung carcinoma (NSCLC) representing lung adenocarcinoma cells (A549 cells). We demonstrated that V-AgNPs interact with A549 cells and exert potent cytocidal action in concentration-dependent manner via mitochondrial depolarization-dependent intrinsic apoptosis pathway. To the best of our knowledge, this is the first report of analysis of V-AgNPs using both 2D and 3D *in vitro* cancer models to understand in-depth mechanistic details behind their cytotoxic action.

## 2 Materials and methods

### 2.1 Materials

The silver nitrate (AgNO_3_), Dulbecco's minimum essential medium (DMEM) with F12 Ham's mixture (1:1 ratio), Dulbecco's phosphate buffered saline (DPBS), paraformaldehyde (Cell culture grade, 4%), sodium cacodylate buffer (pH ∼7.4), sodium azide, paraformaldehyde (Electron microscopy grade, 16%), glutaraldehyde (Electron microscopy grade, 25%), fetal bovine serum (FBS), penicillin-streptomycin mixture, cell counting kit-8 (CCK-8), resazurin, 2′,7′-dichlorofluorescin diacetate (DCFDA) reagent, JC-1 dye, propidium Iodide, tryptic soy broth (TSB) medium, and trypsin were purchased from Sigma Aldrich (Merck), Denmark. Epidermal growth factor (EGF), CellEvent™ Caspase-3/7 Green ReadyProbes™, hoechst, ethidium homodimer II, 4′,6-diamidino-2-phenylindole (DAPI) were purchased from ThermoFisher Scientific, Denmark. All the primary and secondary antibodies; namely, anti-BAX antibody, anti-BCL-2 antibody, anti-β-actin antibody, anti-rabbit-IgG antibody were purchased from Cell Signaling Technology, Denmark. BIOFLOAT™ solution was purchased from faCellitate, Germany. CellTiter-Glo® reagent was purchased from Promega, Denmark. MycoZap™ Plus-PR (10 × 1 ml) was purchased from Bionordika A/S, Denmark. All cell culture vessels such as flasks, well-plates were purchased from Corning Inc., Denmark. Human lung adenocarcinoma cell line (A549 cell line) was purchased from American Type Culture Collection (ATCC, Germany headquarters).

### 2.2 Methods

#### 2.2.1 Synthesis of V-AgNPs from bacteria

The V-AgNPs from *Viridibacillus* culture were synthesized as described previously [20]. Briefly, the bacterial strain was isolated from single colonies that were obtained from the soil samples collected in the fields of Technical University of Denmark (DTU), Denmark. The bacterial isolation procedure followed our previously published protocol [20]. The isolated bacterial strain (99.05% identity with *Viridibacillus arvi* strain LMG 22165) was cultured in shaker incubator at 37 °C for overnight period in 100 mL of TSB medium with shaking speed of 120 rpm. After overnight incubation, the bacterial cells were separated from culture by centrifugation at 8000 rpm for 10 min. To prepare nanoparticles, 1 mM AgNO_3_ was added to cell-free supernatant separated from centrifugation step and kept it in a shaker incubator at 37 °C with speed of 200 rpm for 48 h. The nanoparticle formation was confirmed with the aid of visual inspection (color change) and UV–visible spectroscopy. Then, whole suspension was centrifuged at 14,000 rpm for 15 min to separate formed V-AgNPs, which were then washed thrice with milliQ water. After final washing step, the nanoparticles were suspended again in milliQ water. The concentration of nanoparticle suspension was determined using single particle-inductively coupled plasma mass spectroscopy (sp-ICPMS) before using it for all further experiments.

#### 2.2.2 Characterization of V-AgNPs

V-AgNPs were characterized by dynamic light scattering (DLS), transmission electron microscopy (TEM), and scanning electron microscopy (SEM) coupled with energy dispersive X-ray (EDX) detector. For DLS, the original suspension of V-AgNPs was diluted 20 times (50 μl–1000 μl) with milliQ water and 1 ml of diluted suspension was analyzed using Malvern Zetasizer Nano ZS90 (Malvern, UK) for determining hydrodynamic diameter and polydispersity index (PDI). The zeta potential of the nanoparticles was measured using folded capillary zeta cell provided by Malvern, UK. Further, in order to visualize the V-AgNPs by TEM, a drop of diluted suspension was put on carbon coated copper mesh grid and analyzed using FEI Tecnai T20 G2 TEM at 200 kV voltage. Similarly, for elemental mapping, a drop of original V-AgNPs suspension was put on a carbon tape on an aluminum stub. After overnight drying, the dried nanoparticles were analyzed using Quanta FEG 200 ESEM microscope equipped with EDX detector. Silver content was confirmed by focusing on particular spot having maximum number of nanoparticles.

#### 2.2.3 Conventional 2D cell culture

For 2D culture, we used human lung adenocarcinoma cell line (A549 cell line). Unless otherwise mentioned, for all the experiments, the DMEM with F12 Ham's mixture (1:1) supplemented with 10% FBS, 1x penicillin-streptomycin, and 1x Mycozap antibiotics was used as growth medium. All the 2D cell culture experiments were carried out on the cells from passage number 81–89 (original passage number was 81 when obtained from ATCC) using an incubator maintained at 37 °C with supply of 5% carbon dioxide (CO_2_) gas. Cell culture grade vessels such as tissue culture treated multiwall plates, flasks, dishes were used as per the need of experiments. The detailed methodology for each cell culture experiment is given in respective sections below.

##### 2.2.3.1 Cell viability assay

To check the cell viability in presence of V-AgNPs, the cells grown in T25 flask (∼75% confluence) were trypsinized and seeded in 96-well plate with seeding density of 10000 cells/well. After allowing them to grow in plate for 24 h, the cells were treated with various doses of V-AgNPs (0.2–1.2 μg/ml). Untreated cells were used as control. After 24 h of treatment, the cells were treated with CCK-8 kit. The live cells reduce 2-(2-methoxy-4-nitrophenyl)-3-(4-nitrophenyl)-5-(2,4-disulfophenyl)-2H-tetrazolium monosodium salt) (WST-8) dye in CCK-8 kit with the help of dehydrogenases enzymes to its water soluble formazan derivative (WST-8 formazan) that shows absorption maxima (*λ*_max_) at 450 nm. Thus, followed by 1 h incubation with CCK-8 dye, the absorbance of each well was checked at 450 nm. We also used resazurin dye to rule out interference from V-AgNPs in CCK-8 kit mediated cell viability assay (explained in Result and Discussion section). Briefly, after treating A549 cells with 0.2–1.2 μg/ml V-AgNPs for 24 h, 20 μl of 150 μg/ml resazurin solution was added to each well to make final volume of 100 μl. Non-fluorescent resazurin dye gets converted to fluorescent resorufin (λ_em_: 590 nm) by reductases present in live cells. Thus, the fluorescence intensity remains directly proportional to the number viable cells. Upon incubation for 1 h, the fluorescence readings were measured at 590 nm. In both assays, the cell viability was calculated and normalized with respect to the viability in the control cells. The results were calculated and represented as % mean viability ± standard error (*n* = 6). From these data and using a Matlab based Dr. Fit program (Version 1.042), the median lethal dose (50% cell death) and less lethal and relatively safer dose (25% cell death) were determined and used for further experiments. Throughout the manuscript, these doses have been termed as ‘LD50-2D’ and ‘LD25-2D’ for 50% and 25% cell death in 2D cultured A549 cells respectively.

##### 2.2.3.2 Cell migration assay

The cell migration assay was performed in a 96-well plate using 3D printed cell insert as per published protocol [21]. Briefly, to perform scratch assay, the 3D printed cell inserts were kept in each well (3 inserts per group) and cells were seeded with seeding density of 50000 cells/well. With this density, A549 cells formed the monolayer inside the well around the 3D printed insert in 24 h. After 24 h, the 3D printed inserts were removed. Then, the monolayer of A549 cells was washed twice with DPBS to remove dead cells and/or cell debris and to deprive it of growth factors, it was incubated with serum free DMEM medium for overnight period. After that, the cells were separately treated with serum containing DMEM (termed as ‘Control’ treatment), DMEM supplemented with serum and EGF (termed as ‘EGF’ treatment), and DMEM supplemented with serum, EGF and LD50-2D of V-AgNPs (termed as ‘EGF + V-AgNPs’ treatment). The well-plate was immediately transferred to an incubator mounted with IncuCyte S3 live cell imaging platform (Firmware 20192.4.0.0, GUI Version 2019B Rev2, and Controller Version 2019B Rev 3). The program was set for image capture of the whole well for 2 days with every 2 h interval. The images were collected in 24-bit TIF format. The image analysis was done with our previously developed protocol [22] using a macro script in FIJI (Fiji Is Just ImageJ, Version 1.53t, National Institutes of Health, USA, Java 1.8.0_322 (64 bit)) in ‘batch processing’ format. Additionally, a Matlab-GUI based program called ‘CellTracker’ was also used to determine the directionality and average cell speed as per the instruction of developer [23]. Briefly, the stack of images was treated for ‘*Vignetting Correction*’ and ‘*Automatic Alignment*’ using CellTracker program. Using ‘*Manual Tracking*’ option with ‘*Linear Interpolation (Faster)*’ mode and fixing ‘*Maximum Cell Displacement*’ value to 600 and ‘*Cell Diameter*’ value to 20, we tracked multiple cells at random to generate their X–Y coordinates in each time frame. Finally, using ‘*Statistics*’ option of CellTracker, we determined cell displacement, average cell velocities, and cell movement directions.

##### 2.2.3.3 Reactive oxygen species (ROS) quantification

To check if V-AgNPs induce ROS in 2D cultured A549 cells, first, the cells were seeded in 96-well plate with cell seeding density of 20000 cells/well. The cells were treated with LD25-2D and LD50-2D doses of V-AgNPs for 24 h. After 24 h, the old medium was removed and cells were incubated with 25 μm DCFDA dye solution for 30 min at 37 °C in the incubator with 5% CO_2_ supply. DCFDA dye gets converted it to its carboxylated anion form by cellular esterases, which in presence of ROS metabolizes to 2′,7′-dichlorofluorescein (DCF) and shows green fluorescence [24]. After 30 min, fluorescence intensities were recorded for all samples at emission wavelength of 495 nm. Untreated cells served as control for this experiment. Final results of absolute fluorescence intensities were represented as mean ± standard error (*n* = 6).

##### 2.2.3.4 Mitochondrial depolarization assay

Mitochondrial depolarization was analyzed using JC-1 dye. A549 cells were seeded in 24-well plate at seeding density of 50000 cells/well. After 24 h, the cells were treated with LD25-2D and LD50-2D doses of V-AgNPs. Untreated cells were used as control. After 24 h incubation with V-AgNPs, old medium was removed. 1 ml new medium containing JC-1 dye solution was added to each well. Working concentration of JC-1 dye was kept to 2 μg/ml. After incubating cells with JC-1 dye for 30 min, images were captured at green emission (*λ*_max_: 520 nm) and at red emission (*λ*_max_: 596 nm). All images were captured using Leica DM4000 microscope with 20X magnification.

##### 2.2.3.5 Nuclear condensation assay

Nuclear condensation was analyzed using hoechst dye. A549 cells were seeded in 24-well plate at seeding density of 50000 cells/well. After 24 h, the cells were treated with LD25-2D and LD50-2D doses of V-AgNPs. After 24 h incubation with V-AgNPs, old medium was removed. The untreated cells (control) and treated cells were washed twice with DPBS. Then using 4% paraformaldehyde, the cells were fixed for 15 min at room temperature. Using NucBlue™ Live ReadyProbes™ reagent (2 drops/1 ml of DPBS), the cells were stained. After staining, the cells were washed twice with DPBS and using Leica DM4000 microscope the imaging was performed at 20X magnification.

##### 2.2.3.6 Cell cycle analysis

To check if V-AgNPs affect cells from particular phase of cell cycle, we employed flow cytometry. In case of 2D cultured A549 cells, the cells were seeded in 6-well plate at seeding density of 1 × 10^5^ cells/well. After 24 h incubation, these cells were treated with LD25-2D and LD50-2D doses of V-AgNPs for 24 h. After 24 h, the old medium was removed. Cells were washed with DPBS and trypsinized. Trypsinized cells were washed thrice with DPBS and fixed with 1 ml of 70% ethanol immediately. Then, to these ethanol fixed cells, 50 μl of PI (Stock concentration 1 mg/ml) was added. After incubating fixed cells with PI for 10 min in dark at room temperature, the suspension was centrifuged at 600 g for 10 min. Further, fixed and stained cells were washed once with DPBS and resuspended in 1 ml of DPBS. This suspension was then subjected to flow cytometry using NovoCyte Quanteon flow cytometer (Agilent Technologies, Denmark). Untreated cells served as control for this experiment. For 3 replicates of each group, 10000 cells (per replicate) were analyzed by flow cytometer. By selecting viable cells (excluding cell debris that appear near X–Y origin of event density graph) and narrowing down to single events (singlets) using appropriated gates, PI stained cells were focused. Using Novoexpress software and its cell cycle analysis module, the histograms of PI stained cells in each group were analyzed. The data in terms of % cell in each cell cycle phase (G0/G1-, S-, and G2/M − phases) were obtained. The results were calculated from these data and represented as % mean ± standard error (*n* = 3).

##### 2.2.3.7 Western blot assay

To check the expression of an anti-apoptotic protein (B-Cell Lymphoma-2 protein (BCL-2)) and a pro-apoptotic protein (BCL-2 associated X protein (BAX)), 5 × 10^6^ cells were seeded in Corning 150 mm cell culture dish (Corning® 150 mm TC-treated Culture Dish - 430599) and allowed to grow for 2 days. After 2 days, cells were treated with LD50-2D for 24 h. After 24 h, cells were scrapped off using a sterile scrapper and washed thrice with ice-cold DPBS. After washing, the cell pellet was lysed for 30 min in 1 ml ice-cold radioimmunoprecipitation assay (RIPA) buffer supplemented with protease inhibitors. The protein concentration was determined using Bradford's reagent with bovine serum albumin (Stock: 2 mg/ml) as standard. 70 μg of total protein from lysate of untreated and V-AgNPs treated cells was loaded in each well and gel electrophoresis was performed using stain-free 4–20% precast polyacrylamide gel at 100 V for 50 min. 15 μl of Precision Plus protein dual color standard was loaded to each gel as reference standard for molecular weight. The protein bands were then transferred to Poly(vinylidene fluoride) (PVDF) membrane (0.2 μm pore size, low fluorescence) using iBlot 2 transfer device at 25 V for 6 min. The membrane was then blocked using 5% skimmed milk mixed in Tris-buffered saline with 0.1% Tween® 20 detergent (TBST) buffer for 12 h. After blocking, membrane was washed thrice with TBST buffer (10 ml/wash). Then membrane was incubated with 1:1000 dilution of BCL-2 (124) mouse mAb (Cell signaling technology #1507) and Bax (2D2) mouse mAb (Cell signaling technology #89477) as well as with 1:10000 dilution of β-Actin (8H10D10) mouse mAb (Cell signaling technology #3700). The primary antibodies were diluted in 5% skimmed milk mixed in TBST buffer. The incubation was performed at room temperature for 3 h. After 3 h, the membrane was washed again three times with TBST buffer (10 ml/wash). Then membrane was incubated with 1:2500 dilution of secondary antibody (Anti-mouse IgG, HRP-linked Antibody, Cell signaling technology #7076) at room temperature for 2 h. After this incubation the membrane was washed again three times with TBST buffer (10 ml/wash). Finally, using Pierce™ ECL western blotting substrate (Thermo-Fisher Scientific #32106), the blot was developed. Imaging was performed using Amersham™ Imager 600 (AI600, GE Healthcare Life Sciences). For BCL-2, BAX and β-Actin, separate blots were developed. Quantification of western blots was done using GelAnalyzer software (Version 19.1).

##### 2.2.3.8 Caspase 3/7 assay

In order to confirm the V-AgNPs mediated apoptosis, we analyzed caspase 3/7 activation in 2D cultured A549 cells using fluorescence microscopy. For 2D cultured cells, 1 × 10^5^ cells were seeded per well in a 6-well plate. After treating them with LD25-2D and LD50-2D doses of V-AgNPs for 24 h, 2 drops of CellEvent™ Caspase-3/7 Green ReadyProbes™ Reagent (ThermoFisher Scientific, Denmark) per ml of cell culture medium was added to plate. Cells were incubated with this reagent for 30 min at 37 °C in the incubator with 5% CO_2_ supply. ReadyProbe™ Reagent that has a DNA binding dye covalently linked to DEVD peptide. Active form of caspase 3/7 breaks this covalent bond leading to attachment of dye to DNA which yields bright green fluorescence. After 30 min, imaging was done using fluorescence microscope (Leica DM 4000B) using 10X magnification. DAPI was used as counterstain for cell nuclei. Untreated cells served as control for this experiment.

#### 2.2.4 3D cell culture to prepare A549 spheroids

For 3D culture, we used same cell line (A549 cell line). To prepare A549 spheroids, we used a 96-well plate with V-bottom. The inner surface of well was converted to biocompatible ultra-low attachment surface using 50 μl of Biofloat™ Flex coating solution as per manufacturer's protocol. After removing this solution and brief drying, 100 μl DMEM supplemented with 10% FBS was added to each well and the well plate was kept in incubator for conditioning. The cells grown to ∼75% confluency in T75 flask were trypsinized and seeded with starting cell density of 1200 cells/well in aforementioned conditioned well plate. After cell seeding, the well plate was centrifuged at speed of 500 g for 2 min to bring all cells down in V-bottom in the vicinity of each other. Additional cell culture medium was added to make final volume to 150 μl/well. The spheroids were grown for 5 days and used for further assays. For all the experiments, the DMEM with F12 Ham's mixture (1:1) supplemented with 10% FBS, 1x penicillin-streptomycin, and 1x Mycozap antibiotics was used as growth medium. All the 3D cell culture experiments were carried out on the cells from passage number 81–89 in an incubator maintained at 37 °C with supply of 5% CO_2_ gas. The detailed methodology for each 3D spheroid culture experiments is given in respective sections below.

##### 2.2.4.1 A549 spheroid growth

A549 cells were seeded in each well of biocompatible V-bottom ultra-low attachment 96-well plate at starting seeding density of 1200 cells/well. To monitor the spheroid growth and its kinetics, immediately after cell seeding, the plate was kept in an incubator equipped with IncuCyte S3 live cell imaging platform (Firmware 20192.4.0.0, GUI Version 2019B Rev2, and Controller Version 2019B Rev 3). Using spheroid module of the software, brightfield images were captured at 10X magnification at every 12 h. To determine the diameter of the spheroids, the images were analyzed using FIJI software (Fiji Is Just ImageJ, Version 1.53t, National Institutes of Health, USA, Java 1.8.0_322 (64 bit)) and the data were represented as mean ± standard error (*n* = 6).

##### 2.2.4.2 A549 spheroid viability assay

The A549 spheroids grown for 5 days in ultra-low attachment V-bottom 96-well plate were used for viability study. 5 days-old spheroids were treated with 4 different concentrations of V-AgNPs (2 μg/ml, 4 μg/ml, 8 μg/ml, and 16 μg/ml) for 24 h. After 24 h, the viability of spheroids was analyzed using CellTiter-Glo® reagent as per manufacturer's protocol. Briefly, first, from each well containing untreated or V-AgNPs treated spheroid, the old medium (150 μl: with or without nanoparticles) was replaced with fresh 100 μl of new medium. To this, 100 μl of CellTiter-Glo® reagent was added and mixed vigorously using multichannel pipette for 5 min. After mixing, the well plate was kept at room temperature in dark for 30 min. CellTiter-Glo® reagent lyse the spheroids to release adenosine triphosphate (ATP) molecules. The reaction between these ATP molecules, the substrate, and the luciferase enzyme yield luminescence signal. As the number of ATP molecules are directly proportional to the number of live cells, the luminescence signal yields the number of viable cells from spheroids. This luminescence was then measured with settings of ‘auto gain’ and 50 ms as ‘integration time’ per well. The luminescence readings from 12 spheroids were measured for each group. The spheroid viability was calculated and normalized with respect to the viability in the control cells. The results were calculated and represented as % mean viability ± standard error (*n* = 12). Similar to 2D cell viability assay, from these data and using a Matlab based Dr. Fit program (Version 1.042), the median lethal dose (50% spheroidal cell death) and less lethal, relatively safer dose (25% spheroidal cell death) were determined and used for further experiments. Throughout the manuscript, these doses have been termed as ‘LD50-3D’ and ‘LD25-3D’ for 50% and 25% cell death in A549 spheroids respectively.

##### 2.2.4.3 Effect of V-AgNPs on the growth of A549 spheroids (In vitro tumor progression model)

To assess the effect of nanoparticles on growth of spheroids, we seeded 1200 cells per well of ultra-low attachment V-bottom 96-well plate. On the day of cell seeding (i.e. at time (t) = 0), V-AgNPs at LD25-3D concentration were added to each well. The shape, size and cell death were observed with fluorescence microscopy using hoechst and ethidium homodimer II dyes. Every day (starting from t = 0), 20 μl of hoechst solution (Stock concentration: 1 mg/ml) and 20 μl of ethidium homodimer II solution (Stock concentration: 5.2 μg/ml) were added to each well containing spheroid forming cells and incubated at 37 °C in the incubator with 5% CO_2_ supply for 1 h. After 1 h, imaging was done using fluorescence microscope at blue emission (*λ*_max_: 470 nm) and red emission (*λ*_max_: 617 nm). 8 replicates were used for each group per day. The intensity of blue and red fluorescence was measured using FIJI software (Fiji Is Just ImageJ, Version 1.53t, National Institutes of Health, USA, Java 1.8.0_322 (64 bit)) and the data were represented as mean ± standard error (*n* = 8). Untreated growing spheroids were used as control in this experiment.

##### 2.2.4.4 Effect of V-AgNPs on the diameter of A549 spheroids (In vitro tumor size model)

To assess efficacy of V-AgNPs on matured 5-days old spheroids, we first prepared A549 spheroids as per the protocol given in method 2.2.4. 5-days old spheroids were then incubated with various concentrations of V-AgNPs that were below LD50-3D dose for 3 days. The images of untreated and V-AgNPs treated spheroids were captured before and after treatment using brightfield microscopy (Leica Microsystems, Germany). From the images, the diameters of A549 spheroids (on 5th day and 8th day) were measured using FIJI software (Fiji Is Just ImageJ, Version 1.53t, National Institutes of Health, USA, Java 1.8.0_322 (64 bit)). The results were represented in terms of mean ± standard error of diameters before and after treatment of V-AgNPs (*n* = 24).

##### 2.2.4.5 Cell cycle analysis

For A549 spheroids, same procedure was followed as described in method 2.2.3.6. Briefly, 24 spheroids were used for each group (Untreated (control), LD25-3D treated and LD50-3D treated spheroids). After treating the spheroids with V-AgNPs for 24 h, the spheroids were washed once with DPBS and subjected to trypsinization. After trypsinization, same aforementioned procedures for cell fixation, cell staining, and final washes of stained cells were followed. 30000 cells per replicate per group were analyzed using NovoCyte Quanteon flow cytometer. Same procedure was followed for cell cycle analysis and results were reported as % mean ± standard error (*n* = 24).

##### 2.2.4.6 Caspase 3/7 assay

In case of A549 spheroids, after treatment with LD25-3D dose of V-AgNPs for 24 h, 20 μl of CellEvent™ Caspase-3/7 Green ReadyProbes™ Reagent was added in each well and incubation was continued for 1 h. After 1 h, the imaging of 3 spheroids per group was performed using 4X magnification (‘whole well analysis’ module) of IncuCyte S3 live cell imaging platform (Firmware 20192.4.0.0, GUI Version 2019B Rev2, and Controller Version 2019B Rev 3). The line profile for the green fluorescence intensity activated caspase 3/7 was drawn using FIJI software (Fiji Is Just ImageJ, Version 1.53t, National Institutes of Health, USA, Java 1.8.0_322 (64 bit)).

##### 2.2.4.7 TEM of 3D spheroids

We used TEM to analyze ultrastructural changes in A549 spheroids after V-AgNPs treatment. 5-days old spheroids were treated with V-AgNPs at LD50-3D concentration for 24 h after 24 h, the untreated (control) as well as nanoparticles treated spheroids were washed twice with DPBS and fixed using Karnovsky fixative at 4 °C for overnight period. The samples were then negatively stained with 1% osmium chloride (diluted in 0.1 M sodium cacodylate buffer) at room temperature for 30 min. 3 washes were given to remove excess salt and then samples were treated with 1% tannic acid solution at room temperature for 30 min. After triple wash cycle, sample dehydration was performed in stepwise manner in 30%, 50%, 70%, 85%, 95%, and 100% ethanol solutions (5 min/step) at room temperature. The samples were then embedded in resins for cutting into micro-sections. These micro-sections were then analyzed for any ultrastructural changes using The Thermo Scientific™ Talos L120C transmission electron microscope.

#### 2.2.5 Statistical analysis

Statistical analysis was done using GraphPad Prism software (Version 9.4.0 (673), GraphPad LLC). For all the experiments, results were reported as mean ± std. error unless stated otherwise. For cell viability assay, spheroid viability assay, caspase 3/7 activation assay, ROS assay, mitochondrial depolarization assay, and assay estimating efficacy of V-AgNPs in spheroidal growth, we used one-way ANOVA test. *Post-hoc* tests were also employed along with one-way ANOVA to confirm the statistical findings in multiple comparisons. For analyzing the results of cell migration assay, cell cycle analysis assay, and effect of V-AgNPs on spheroids, we used two-way ANOVA test with multiple comparisons. For western blot analysis, we used unpaired *t*-test. All the statistical tests were performed at confidence interval of 95% (i.e., with level of significance of 0.05). The data, for which statistical analysis showed *p*-value lesser than 0.05, were considered significantly different from each other.

## 3 Result and discussion

### 3.1 The Viridibacillus enriched culture based green method produce highly stable V-AgNPs in reproducible manner

**Fig. 1:**
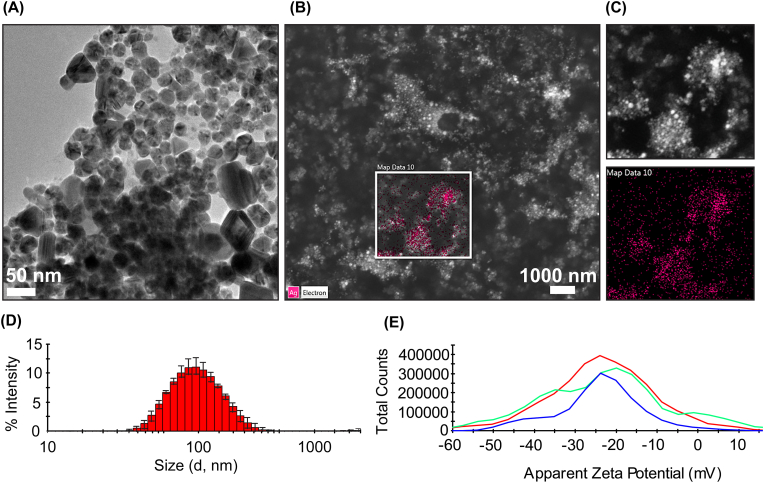
Synthesis and characterization of *Viridibacilli* enriched culture derived silver nanoparticles (V-AgNPs). **(A)** Transmission electron micrograph showing nearly spherical nanoparticles with seldom presence of cuboidal and triangular particles within the size range of 20–50 nm. **(B and C)** Scanning electron micrograph of V-AgNPs in which a spot was focused for analyzing elemental composition. The pink colored spot overlapping bright white spots in electron micrograph confirmed the presence of silver element. **(D and E)** Dynamic light scattering analysis of V-AgNPs showed average hydrodynamic diameter of around 168.1 ± 1.1 nm and average zeta potential of about −23.8 ± 0.3 mV. (For interpretation of the references to colour in this figure legend, the reader is referred to the Web version of this article.)

In order to check the anticancer potential, we synthesized V-AgNPs with previously optimized process parameters [20] (temperature: 37 °C, 200 RPM shaking, and 48 h incubation time) and upon characterization, we found that this method is highly reproducible with respect to size, shape, and yield of the nanoparticles (Fig. 1A–E). The transmission electron micrograph showed nearly spherical particles with minor fraction in cuboidal, or triangular shape (Fig. 1A). The V-AgNPs showed 40–50 nm sized particles in TEM image. The SEM coupled with EDX detector showed presence of silver confirming reduction and conversion of silver salt into AgNPs (Fig. 1B and C). DLS analysis of V-AgNPs showed that the hydrodynamic diameter of particles was 168.1 ± 1.1 nm with PDI of 0.212 ± 0.02. DLS also reflected that 90% of particle population was below 250 nm (Fig. 1D). The zeta potential of V-AgNPs was in the range of −23.8 ± 0.3 mV (Fig. 1E). All these results corroborated well with our previously published data [20]. The particle size and zeta potential play vital role in any biomedical application of the nanoparticles [[25], [26], [27]]. Yet, there is no gold standard to define the optimum nanoparticle size in drug delivery applications. Specifically for anticancer applications, vast amount of literature shows that sizes ranging from 50 to 200 nm have produced desirable anticancer effect [26,28,29]. Along with surface properties, size of the nanoparticles also plays important role in their endocytosis in the cancerous cells. Recently, it has been stated that the nanoparticles with hydrodynamic diameter below 200 nm get internalized via either clathrin-coated pit-mediated endocytosis (CME), or fast endophilin-mediated endocytosis (FEME), or clathrin-independent carrier (CLIC)/glycosylphosphatidylinositol-anchored protein enriched early endocytic compartment (GEEC) mediated endocytosis pathway [26,28]. As our V-AgNPs showed hydrodynamic size well within this range, it is possible that these mechanisms could contribute to their success in getting internalized and show potent anticancer action. Another recent study suggest that the metal nanoparticles having bigger size contribute significantly to the interaction and damage of lipid bilayer; whereas nanoparticles with lower size get internalized without damaging it [30]. With same principle, it is possible that the sub-200 nm hydrodynamic size of our V–AgNPS could contribute to the endocytosis process, membrane damage, and overall anticancer action. The particle size of AgNPs is also associated with their fate in terms of their excretion or accumulation and nanotoxicity. Using animal models, it has been shown that ultrasmall AgNPs remain in the body for around 28 days that could lead to accumulation and side-effects. But, the AgNPs with size greater than 50 nm show optimum therapeutic efficacy and get excreted faster, thus showing considerable biosafety and biocompatibility [31,32]. Our V-AgNPs had a size of around 40–50 nm (in dried form by TEM) and hydrodynamic size of 168.1 ± 1.1 nm (in suspension form by DLS) which qualifies them for biosafety and easy excretion without accumulation in the body. So with such results, we could predict that the chances of off-target effects would be very low.

After synthesis and characterization of V-AgNPs, we used A549 cell line in 2D and 3D culture format for analyzing their anticancer potential. For the same, when the aqueous suspension of nanoparticles was diluted with cell culture medium, no aggregation of V-AgNPs was observed during dilution, which suggests that V-AgNPs have excellent stability in mammalian cell culture medium. This in turn also suggest that, over the experimental duration, the action of V-AgNPs would be only due to their nano-form and not the aggregates. In our previous report, we have shown their stability in aqueous suspension as well as in bacteriological medium over longer period [20]. Excellent stability in biological media is one of the essential requisites of for all colloidal nanomaterials in terms of their shelf-life as well as their intended biomedical applications. Our V-AgNPs fulfilled this criterion owing to their biological corona formed on their surface during synthesis. The success behind such tremendous stability could be attributed to the negative zeta potential of the V-AgNPs offered by this biological corona. The negative zeta potential helps the nanoparticles to repel each other when comes into vicinity. It leads to prevention of their aggregation and it also helps in maintaining the Brownian motion [25]. Moreover, it has been shown that zeta potential is one of the few properties that control nanoparticle-cell interactions (also termed as nano-bio interactions) and endocytosis of the nanoparticles [26,27].

### 3.2 The V-AgNPs show dose dependent cytotoxicity towards 2D and 3D cultured A549 cells

**Fig. 2:**
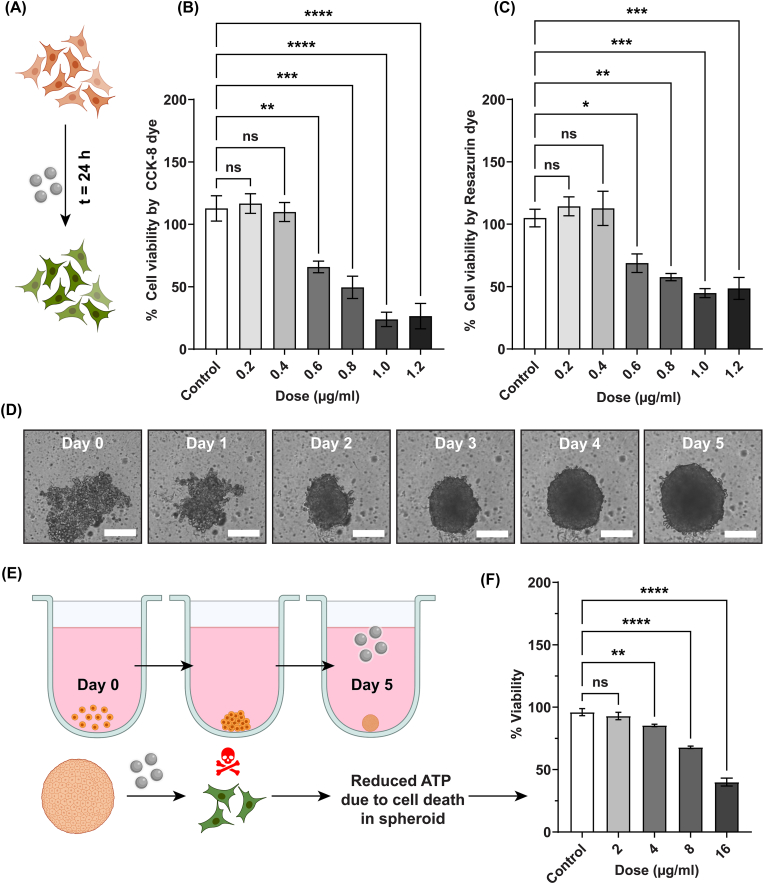
Effect of V-AgNPs on the cell viability in 2D cultured A549 cells and 3D A549 spheroids. **(A)** Schematic shows that 2D cultured A549 cells were treated with various concentrations of V-AgNPs for 24 h and then using two different assays, percentage cell viability was determined. Viable (Live) and non-viable (dead) cells are represented by orange and green colors, respectively. **(B and C)** The bar graphs show viability of A549 cells determined using CCK-8 cell viability kit and resazurin after incubating cells with 0.2–1.2 μg/ml of V-AgNPs. Each bar represents mean ± std. error of 6 replicates (*n* = 6). **(D)** The brightfield time lapse images showing growth of 3D *in vitro* model of lung cancer i.e. A549 spheroids from 0^th^ day (day of cell seeding) to 5th day. (Scale bar in the images: 200 μm) **(E)** The schematic shows growth of spheroids till 5th day and addition of V-AgNPs at various concentrations in 5-days old A549 spheroids. After incubating spheroids for 24 h, the spheroidal cell viability was measured using Cell titer glo 3D® reagent. **(F)** The bar graph shows spheroidal cell viability in presence of 2–16 μg/ml V-AgNPs and each bar represents mean ± std. error of 12 replicates (*n* = 12). The asterisks in the bar graphs represent significantly different observations (One-way ANOVA test: * p-value<0.05, **p-value<0.01, *** p-value<0.001, **** p-value<0.0001). (Abbreviations – t: time, h: hours, ATP: Adenosine triphosphate, ns: not significant) (Schematics were created with BioRender.com). (For interpretation of the references to colour in this figure legend, the reader is referred to the Web version of this article.)

As shown in the schematics (Fig. 2A), first we sought to check the cytotoxic effect of V-AgNPs in 2D cultured A549 cells. Upon incubation of cells with various concentrations of V-AgNPs (0.2–1.2 μg/ml) for 24 h, we used CCK-8 cell viability kit for determination of number of viable live cells based on the light intensity at 450 nm [33]. In this assay, no significant differences in percent viability were seen in cells treated with 0.2 μg/ml and 0.4 μg/ml V-AgNPs compared to the untreated control (one-way ANOVA, p-value >0.05) (Fig. 2B). All higher concentrations (0.6–1.2 μg/ml) showed a significant reduction in percent cell viability (one-way ANOVA, p-value <0.05). From these data, the median lethal dose (LD50) was found to be in the range of 0.8–1.0 μg/ml (Fig. 2B). However, the V-AgNPs show surface plasma resonance band in the range of 400–500 nm, where the CCK-8 shows maximum excitation. Hence, to rule out any possible interference and to confirm the findings made with the CCK-8 kit viability assay, we also used resazurin dye for determination of cell viability [34]. Resazurin assay showed a significant reduction in cell viability for the cells treated with V-AgNPs in the concentration range of 0.6–1.2 μg/ml (one-way ANOVA, p-value <0.05), and LD50 in the range of 0.8–1.0 μg/ml (Fig. 2C). For accurate determination of LD50, we employed a Matlab GUI-based Dr. Fit program (Version 1.042) [35]. Using this program, we observed that monophasic standard Hill's equation was the best fit for our cell viability data. The LD50 determined from this curve fitting method was equal to 1 μg/ml (Supporting information, Figure S1 and S2). Extensive review of green AgNPs has shown that in A549 cell line, the AgNPs obtained from Sacred fig leaves extract and E-coli enriched culture medium show LD50 of around 2 μg/ml and 40 μg/ml, respectively [19]. The lower LD50 of our V-AgNPs (as compared to what shown in the literature) renders them more potent as compared to other reported green AgNPs. Due to such low LD50, the probability of unpredictable side-effects of our V-AgNPs is low. This observation further strengthens the biocompatibility and biosafety of our nanoparticles as mentioned in the result section 3.1. In order to deduce dose dependent activity, we also determined relatively less lethal dose (the one that shows 25% cell death). We termed both these doses as LD50-2D and LD25-2D in rest of the manuscript for simplification.

Even though the biochemical features of 2D cultured cancer cell lines mimic that of *in vivo* tumor cells, their microenvironment is far different from the actual tumor microenvironment [36]. In that sense, 3D cultured spheroid is a more accurate cancer model. 3D grown spheroids show distinct zones of proliferative cells, quiescent cells, apoptotic/necrotic cells just like an *in vivo* tumor. Apart from cellular zones, 3D grown spheroids also show a gradient for oxygen, nutrients and pH, similar to that of a tumor microenvironment [36]. Furthermore, the latest guidelines of the European Union also recommend to reduce, to refine, and to replace (3-Rs) experiments using animals whenever possible [37,38]. Hence, the use of 3D spheroidal models has become an obvious choice for studying cancer biology as well as preliminary evaluation of effectiveness of cancer therapeutics [36]. With this purpose, we used 3D spheroids prepared from same A549 cell line (A549 spheroids) to test our nanoparticles.

As shown in Fig. 2D, we cultivated A549 spheroids for 5 days with starting cell density of 1200 cells/well. The growth kinetics (Supporting information, Figure S3) showed linear rising trend and the diameter of spheroids reached to 539 ± 23 μm on 5th day. The cell mass acquired distinct spheroidal shape on 3rd day of cultivation. Previously it has been shown that, A549 cells upon with the seeding density of 5000 form spheroid within the time span of 3–5 days [[39], [40], [41]]. Within this time span, A549 spheroids showed strong cell-cell junctions and apoptotic cells in the core region [41]. Therefore, we selected spheroids of 5 days age for viability experiment. As 3D grown spheroids are a compact mass of cells, one can expect only negligible cytotoxic effects of median lethal dose determined against 2D cell culture (LD50-2D). The simple reason for this is the exposure surface. In 2D cultured cells, every cell is exposed to nanoparticles whereas in case of spheroids, only the outer peripheral region is exposed. Additionally, after exposure, the AgNPs enter a 2D cultured cell easily by crossing one plasma membrane. However, in case of 3D spheroids, nanoparticles need to traverse through several cells (transcytosis) to enter deeper layers of spheroids. This is why higher concentration of nanoparticles is needed to observe significant toxicity in 3D spheroidal models. Moreover, higher rate of proliferation within compact dense cellular mass may actually overcome the cytotoxic action of nanoparticles at lower concentrations. Therefore, we decided to check cytotoxicity of V-AgNPs in A549 spheroids at higher concentrations. To check dose dependent effect of V-AgNPs, we selected concentrations in geometric progression range starting from 2 times to 16 times higher than LD50-2D (i.e., 2–16 μg/ml). As shown in the schematics (Fig. 2E), the V-AgNPs were added to spheroidal cultures on 5th day and incubation was continued for 24 h. Using CellTiter-Glo® 3D cell viability kit, the viability of untreated (control) and V-AgNPs treated spheroids was determined [42]. Based on this assay and data obtained from it (Fig. 2F), we observed that no significant reduction in spheroid viability (one-way ANOVA, p-value >0.05) at 2 μg/ml concentration. However, V-AgNPs within 4–16 μg/ml concentration range did manage to reduce viability significantly (one-way ANOVA, p-value <0.05) in dose dependent manner. In Dr. Fit program, monophasic standard Hill's equation was the best fit for the observed reduction in A549 spheroidal viability. From this fit, we calculated 13 μg/ml as LD50 for A549 spheroidal cultures (Supporting information, Figure S4). Similar to the analysis of 2D cultured A549 cells, we also determined relatively less lethal dose (the one that shows 25% cell death) for A549 spheroids for analyzing dose dependent activity. We termed both these doses as LD50-3D and LD25-3D in rest of the manuscript for simplification.

To summarize, the V-AgNPs showed dose dependent cytotoxicity in both 2D cultured A549 cells as well as 3D cultured A549 spheroids. In A549 cells, LD25-2D and LD50-2D were 0.6 μg/ml and 1 μg/ml, respectively; whereas LD25-3D and LD50-3D in A549 spheroids were 8 μg/ml and 13 μg/ml, respectively.

### 3.3 V-AgNPs exert tumor progression suppressing as well as tumor size reducing activities in A549 spheroidal models

**Fig. 3:**
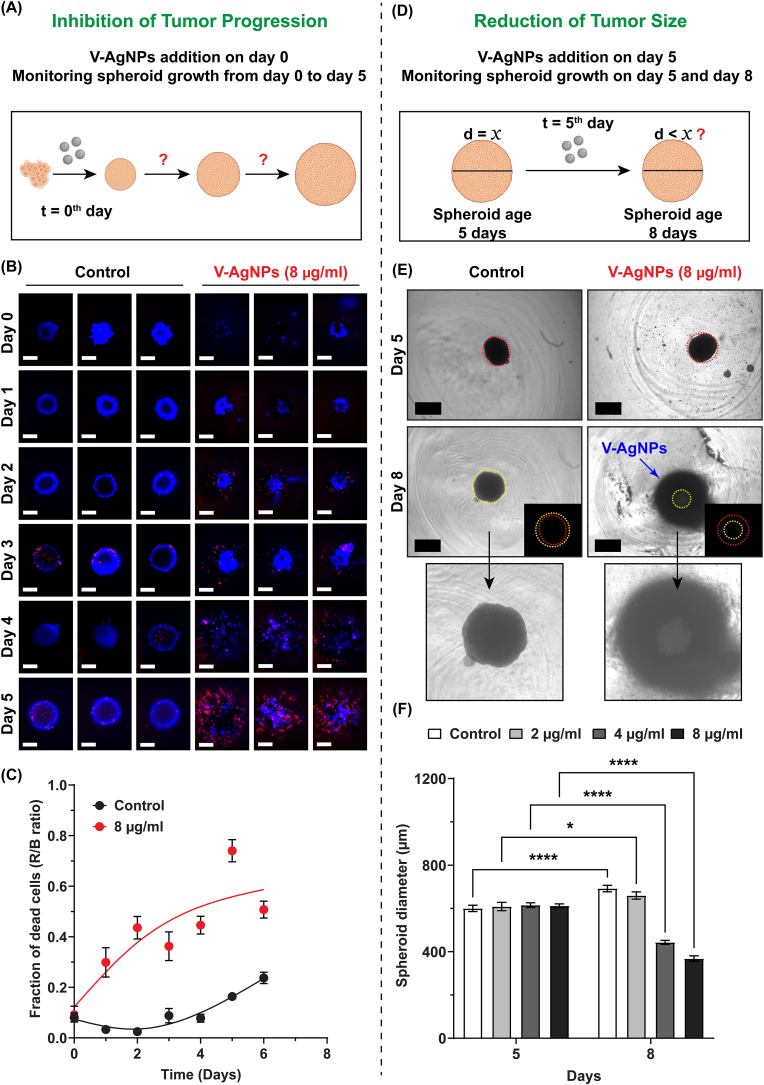
Effect of V-AgNPs on *in vitro* 3D tumor progression model and 3D tumor size model. **(A)** Schematic and description show establishment of *in vitro* tumor progression model in which the A549 cells were seeded (day 0) and on the same day V-AgNPs were added at relatively safer concentration (LD25-3D). The spheroid growth was monitored for the shape and live-dead cell fractions till day 5. Untreated A549 cells i.e. cells forming spheroids in absence of V-AgNPs served as control for this experiment. **(B)** Representative images of spheroid forming cells (either in absence or presence of V-AgNPs) stained with hoechst and ethidium homodimer II. For each group (control and V-AgNPs treated) total 8 spheroids were imaged per day (*n* = 8). Images for all replicates for all days have been given in supporting information, figure S5. Images for only 3 replicates are given here. (Scale bar in the images: 200 μm) **(C)** The graph shows the dead cell fraction (ratio of red fluorescence to blue fluorescence) for untreated and V-AgNPs treated spheroids on each day and each point represents mean fraction ± std. error for 8 replicates (*n* = 8). The line has been drawn using spline fitting function to represent the mean trend of rise of dead cell fraction. **(D)** Schematic and description show establishment of *in vitro* tumor size model in which the A549 cells were seeded (day 0) and grown till day 5. Then 5-days old spheroids were treated with 2–8 μg/ml concentration of V-AgNPs (the highest dose here is LD25-3D). The diameters of spheroids were measured before (Day 5) and after the treatment (Day 8). Untreated A549 spheroids served as control for this experiment. **(E)** Representative brightfield images for untreated and 8 μg/ml V-AgNPs treated cells on day 5 and day 8. The red and yellow dotted lines represent the diameters of spheroid before and after the V-AgNPs treatment, respectively (Scale bar in the images: 500 μm). The inset in the images shows differences in diameter before and after the treatment. The V-AgNPs can be seen around the spheroids as shown by blue arrow in 8th day V-AgNPs treatment image. The third row shows zoomed-in image of untreated and V-AgNPs treated spheroids. The brightfield images for all treatment groups (2–8 μg/ml) are given in supporting information, figure S6. **(F)** The bar graph shows the inter-day comparison of diameter of untreated (control) and 2–8 μg/ml V-AgNPs treated spheroids and each bar represents mean diameter ± std. error for 25 replicates (*n* = 25). The intra-day comparison is given in supporting information, figure S6. The asterisks in the bar graph represent significantly different observations (One-way ANOVA test: * p-value<0.05, **p-value<0.01, *** p-value<0.001, **** p-value<0.0001). (Abbreviations – V-AgNPs: *Viridibacilli* derived silver nanoparticles, t: time, d: diameter, R/B ratio: ratio of Red and Blue fluorescence intensities) (Schematics were created with BioRender.com). (For interpretation of the references to colour in this figure legend, the reader is referred to the Web version of this article.)

Using 2D cultured cells, it is impossible to determine whether an anticancer agent suppresses tumor progression and/or tumor size. Such information is usually deduced using animal models which is time consuming, costly, and requires large number of animals and trained personnel [[43], [44], [45]]. Especially for preliminary high throughput screening of anticancer agents, this process is laborious and expensive. As stated earlier, in the wake of 3R policy for welfare of animals used in scientific experiments, using large number in preliminary screening is not ethical and feasible. But emergence of 3D spheroidal models has aided in replacing animal models to extract such important preliminary information about effectiveness of any anticancer agent. Hence, we decided to use A549 spheroids in two different ways. As lung cancer is often characterized by high progression rate [46], it is imperative to check whether V-AgNPs suppress tumor progression. To do that, we developed a simple experiment (Fig. 3A) where we tried to prepare A549 spheroids in presence of LD25-3D of V-AgNPs. LD50-3D was not selected for this experiment as it would impart higher toxicity within experimental duration defeating the purpose of the experiment. The V-AgNPs were added at the concentration of 8 μg/ml to same culture vessel (ultra-low attachment cell culture plate) along with A549 cells on day 0. Then, the spheroid growth was monitored in terms of shape of spheroids as well as dead cell fraction using Hoechst-PI staining. We observed impaired growth of spheroids and increased cell death in presence of V-AgNPs as compared to untreated growing A549 spheroids. As shown in Fig. 3B and supporting information figure S5, the untreated growing spheroids showed oval to nearly spherical shape on 2nd day and completely spherical shape on 3rd day. But in presence of V-AgNPs, A549 cells did not form spheroidal structure on 3rd day. We presume that the A549 cells failed to exhibit cell-to-cell contacts in order to form a tight spheroidal mass and they remained scattered in the well. Furthermore, upon staining with Hoechst and PI dyes, the fraction of dead cells increased with time in presence of V-AgNPs (Fig. 3C). On the other hand, the untreated spheroids grew well within the well and exhibited significantly less dead cell fraction. The lesser yet distinctive dead cell fraction in untreated A549 spheroids denotes inherent cell death due to apoptosis and hypoxia inside cell mass, which again in turn represents successful formation of tumor microenvironment mimicking 3D spheroidal models.

A bioactive agent, to be a successful anticancer candidate, should also possess tumor size reduction property along with tumor progression inhibitory action [47,48]. Same rule applies to AgNPs too. Therefore, in another experiment (Fig. 3D), to determine the impact of V-AgNPs on tumor size, we used 5-days old A549 spheroids. On 5th day, the V-AgNPs were added at different concentrations in the range of 2–8 μg/ml (Fig. 3D) and the sizes of total 25 A549 spheroids (*n* = 25) before and after V-AgNPs treatment were determined from bright-field images. We observed that the size of untreated and 2 μg/ml V-AgNPs treated A549 spheroids increased significantly (one-way ANOVA, p-value >0.05) (Fig. 3E and F). For control untreated spheroids, it rose from 600 ± 74 μm to 691 ± 73 μm; whereas, for 2 μg/ml treated spheroids, it increased from 609 ± 93 μm to 660 ± 80 μm. But 4–8 μg/ml V-AgNPs showed significant reduction in sizes owing to spheroidal cell death (one-way ANOVA, p-value <0.0.5) (Fig. 3E and F). For 4 μg/ml treated spheroids the diameter decreased from 615 ± 53 μm to 440 ± 45 μm and for 8 μg/ml treated spheroids it came down from 612 ± 44 μm to 368 ± 62 μm. These results are consistent with our spheroid viability assay. As shown in Fig. 3E and supporting information figure S6, in the representative brightfield images, the diameters on 5th day (shown by red dotted line) and 8th day (shown by yellow dotted line) have significant differences for 4 and 8 μg/ml treated spheroids. Intra-day comparison of A549 spheroid diameters on 8th day showed significant differences in all treatment groups except untreated and 2 μg/ml treated spheroids (Supporting information, Figure S6).

To summarize, our data (Fig. 3A–F) reflect that V-AgNPs have capability to reduce tumor size after certain concentration (≥4 μg/ml) and once present in the vicinity, they halt the growth of tumor. To the best of our knowledge, this is the first attempt that shows use of such two different experimental models of A549 spheroids for testing efficacy of V-AgNPs and for extracting the data equivalent to actual animal models. Such experiments further justify utility of 3D grown spheroids in the field of cancer biology and cancer therapeutics. These novel *in vitro* tumor progression model and *in vitro* tumor size model can be further extrapolated in future for multicellular steroids to study cellular interactions and signaling pathways underlying cancer biology. Our presented work showing use of these *in vitro* 3D models also represent significant methodological advancement for preliminary testing of anticancer AgNPs. Use of these 3D cell models can be further extended to other anticancer agents for determination of their cytotoxic activity either alone or in combination, in quicker way as compared to laborious and expensive *in vivo* animal studies.

### 3.4 V-AgNPs reduce cell migration of 2D cultured A549 cells even in the presence of growth factor

**Fig. 4:**
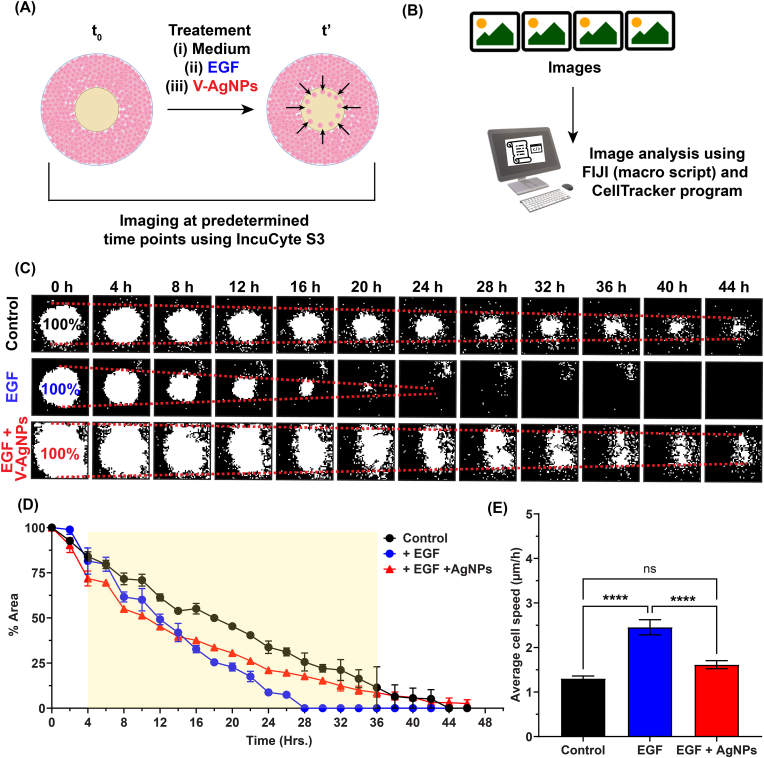
Study of migration of 2D cultured A549 cells in absence and presence of epidermal growth factor (EGF) and V-AgNPs at LD50-2D. **(A)** The schematic shows creation of cell-free area using cell inserts and addition of cell culture medium (control group), EGF (positive control group), and EGF + V-AgNPs (treatment group). Upon these treatments, the time lapse imaging was done to understand A549 cell migration in cell-free area using IncuCyte S3 live cell imaging platform. **(B)** The schematic shows the image analysis pipeline for time lapse images obtained from IncuCyte S3 instrument. The image analysis was done using our previously developed macro script for understanding % cell-free are over time as well as using a Matlab-GUI based CellTracker program for understanding cell migration pattern and migration velocities under various treatments. **(C)** The binary time lapse images obtained after image analysis show percent reduction of cell-free area (white area in the images) due to migration of A549 cells (black area in the images) in presence of only cell culture medium (black, upper row), EGF (blue, middle row), and EGF + V-AgNPs (red, lower row). The % cell-free areas were calculated by considering the cell-free area of 0^th^ h images as 100%. **(D)** The line graph shows the cell migration kinetics under the influence of various treatments, where each line represents mean ± std. error of 3 replicates (*n* = 3). The yellow box indicates the time frame within which significant differences (two-way ANOVA, p-value <0.05) were observed in each treatment. The bar graph showing these differences is given in supporting information, figure S7. **(E)** The bar graph represents average cell migration velocities obtained from CellTracker program and each bar represents mean velocity ± std. error of randomly selected cells (*n* = 10). The cell migration patterns and directionalities in presence of aforementioned treatments are given in the supporting information, figure S8-S10. The asterisks in the bar graph represent significantly different observations (One-way ANOVA test: **** p-value<0.0001). (Abbreviations – V-AgNPs: *Viridibacilli* derived silver nanoparticles, EGF: Epidermal growth factor, t_0_: initial time or start time, t’: time point other that t_0_, h: hours, ns: not significant) (Schematics were created with BioRender.com). (For interpretation of the references to colour in this figure legend, the reader is referred to the Web version of this article.)

Cell migration is a fundamental phenomenon in developmental biology as well as in diseases such as cancer. Cell migration is of prime importance in cancer because of its pivotal role in metastasis [49]. Thus, we checked the cell migration pattern (direction and migration velocity) of the A549 cells treated with V-AgNPs at LD50-2D. As mentioned in the methodology, we used cell inserts [21] to create cell-free area and checked the migration of cells in that area in presence of epidermal growth factor (EGF) and in presence of nanoparticles along with the EGF (EGF + V-AgNPs) (Fig. 4A). The cell migration in absence of these both components served as control for this experiment. After treatments, the migration of cells into the cell-free area was recorded in each sample and using our previously developed macro script [22], the time lapse images obtained from this experiment were then analyzed for measuring the cell-free area (Fig. 4B). We observed that over time, the surface of the cell-free area decreases with varying rates suggesting different proliferative capacities as well as motility of cells under the influence of various treatments (Fig. 4C). As shown in Fig. 4C, the cells treated with the standard cell culture medium (Control group) proliferated and migrated at a steady rate. Cell treated with 25 ng/ml of EGF supplement proliferated and migrated much faster, the cell-free area was filled up completely within 28 h. It is well documented that EGF has a positive impact on cell proliferation and migration, exerted by its binding with EGF receptor (EGFR) [[50], [51], [52]]. Upon binding, it improves cell differentiation, extracellular matrix (ECM) synthesis, causes ECM remodeling, and increases protein synthesis and protein phosphorylation [[50], [51], [52]]. Thus, we used it as a positive control that mimics the natural highly proliferative state of *in vivo* tumor cells growing in presence of various growth factors in the body. When EGF-stimulated cells were treated with LD50-2D of V-AgNPs, we observed a remarkable decrease in the rate of invasion of the cells in the cell-free area (one-way ANOVA, p-value <0.05). We propose that V-AgNPs, owing to their cytotoxic effect, reduce the proliferative and migratory capacity of treated cells. From the cell migration kinetics in the cell-free area, one can conclude that the most significant differences between treated and untreated cells occur in the time frame of 4–34 h (Fig. 4D–Supporting information, Figure S7). At 28 h, the cell-free areas were 25 ± 7% for untreated cells, 18 ± 2% for V-AgNPs treated cells, and 0% for EGF treated cells. We used Matlab based CellTracker program [23] to deduce the cell migration direction and the migration velocity for multiple randomly selected cells. In all the examined cases, cells migrated towards the center of the cell-free zone in forward direction (Supporting information, Figure S8-S10). The converging points for all the migration lines in case of untreated cells and EGF treated cells were between time frames 20–25 and 10–15, respectively. For V-AgNPs treated cells, the lines did not converge even after 25 time frames. These data qualitatively suggest a profoundly different migration behavior of V-AgNPs-treated cells as compared to controls (Untreated and EGF treated cells). Quantitatively, the average cell migration velocity of V-AgNPs treated cells was significantly reduced to 1.6 ± 0.3 μm/h as compared to that of EGF treated cells where velocity was 2.5 μm/h (one-way ANOVA, p-value <0.05) (Fig. 4E).

### 3.5 V-AgNPs drives cell towards DNA damage and mitochondrial depolarization dependent intrinsic apoptosis pathway

**Fig. 5:**
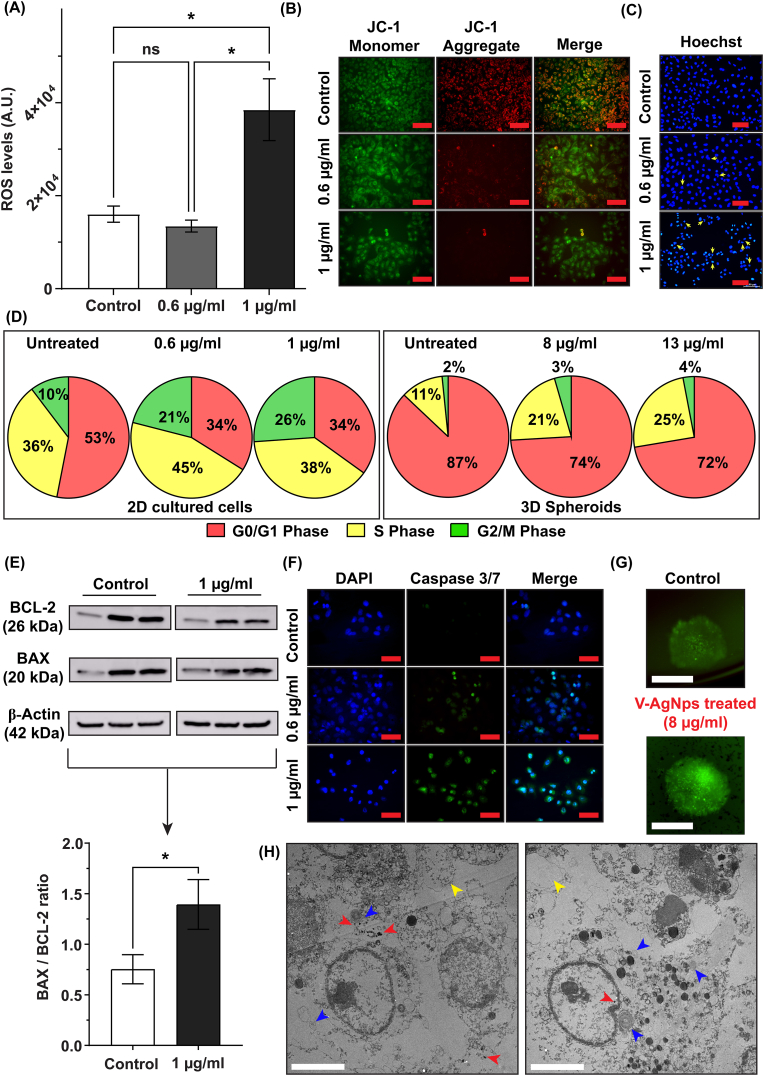
Mechanism of V-AgNPs mediated apoptosis in 2D cultured A549 cells and A549 spheroids deduced from an array of cell and spheroid based assays. **(A)** The bar graph shows levels of reactive oxygen species (ROS) in 2D cultured A549 cells upon treatment with LD25-2D and LD50-2D doses of V-AgNPs, where each bar represents mean ROS ± std. error of 6 replicates (*n* = 6). **(B)** The representative images showed dose dependent mitochondrial depolarization in 2D cultured A549 cells upon treatment with LD25-2D and LD50-2D of V-AgNPs in the form of vanishing red intensity of JC-1 dye aggregates (Scale bar in the images: 100 μm). The green and red intensity of JC-1 dye monomer and aggregates was quantified from images and it is represented as the bar graph in the supporting information, figure S11. **(C)** The representative images of hoechst stained 2D cultured A549 cells showed dose dependent DNA toxicity in the form in condensed and fragmented nuclei (shown by yellow arrows) (Scale bar in the images: 100 μm). **(D)** The pie charts show results of cell cycle analysis obtained from flow cytometry of 2D cultured A549 cells (left) and A549 spheroids (right). Each pie chart shows percentage of cells arrested in G0/G1-phase (red), S-phase (yellow), and G2/M-phase (green) and represents mean percentage values for 10000 cells measured in triplicate (*n* = 3). The histogram, the cell cycle fitting, and inter-group analysis (two-way ANOVA) are given in supporting information, figure S12. **(E)** The image shows western blot analysis for untreated and LD50-2D treated 2D cultured A549 cells for three replicates (*n* = 3). The bar graph underneath the blot images shows significant elevation in the ratio of BAX:BCL-2 expression in V-AgNPs treated cells as compared to control (*t*-test, p-value <0.05). The separate quantification of BCL-2 and BAX protein expression relative to β-actin expression is given in bar graph in supporting information, figure S13. **(F)** The representative images showed dose dependent activation of caspase 3/7 enzymes in 2D cultured A549 cells upon treatment with LD25-2D and LD50-2D of V-AgNPs in the form of increasing green fluorescence intensity of CellEvent™ caspase 3/7 reagent (Scale bar in the images: 50 μm). The quantification of green fluorescence intensity relative to blue fluorescence of DAPI stained nuclei is shown as bar graph in supporting information, figure S14. **(G)** The representative images of control and LD25-3D treated A549 spheroids show activated caspase 3/7 enzymes confirming apoptosis process induced by V-AgNPs. (Scale bar in the images: 200 μm) The line profile given in supporting information, figure S15 confirms that green fluorescence intensity in V-AgNPs treated spheroids is increased and spread over central as well as peripheral regions of spheroidal structure unlike control untreated spheroids. **(H)** Transmission electron micrograph of A549 spheroids confirmed presence of V-AgNPs inside the cells (red arrowheads), mitochondrial toxicity (blue arrowheads), and dead cells (yellow arrowheads) inside spheroids (Scale bar in the images: 5 μm). The asterisks in the bar graph represent significantly different observations (One-way ANOVA test: * p-value<0.05). (Abbreviations – ROS: reactive oxygen species, A.U.: arbitrary units, ns: not significant, 2D: 2-dimensional, 3D: 3-dimensional, BCL-2: B-cell lymphoma-2 protein, BAX: BCL-2 associated X protein, DAPI: 4′,6-diamidino-2-phenylindole, V-AgNPs: *Viridibacilli* derived silver nanoparticles). (For interpretation of the references to colour in this figure legend, the reader is referred to the Web version of this article.)

Over the last two decades, several metallic nanoparticles have been explored for their anticancer potential with or without aid of external components such as ultrasound waves or laser [53]. It has been shown that these nanoparticles induced apoptosis via intrinsic or extrinsic pathway [53,54]. In order to investigate and pin-point exact mechanism of apoptosis caused by V-AgNPs, we performed range of bioassays using A549 cells and A549 spheroids. First, we analyzed the ROS levels in 2D cultured A549 cells using DCFDA dye. In presence of elevated ROS levels, the DCFDA forms green fluorescent DCF [24]. Thus, the ROS levels are directly proportional to the green fluorescence intensity. As shown in Fig. 5A, LD50-2D showed significantly elevated ROS levels in A549 cells as compared to untreated and LD25-2D cells (one-way ANOVA, p-value <0.05). ROS level at LD25-2D remained as low as untreated cells, which suggested that at this dose, the cells were capable to counter the ROS toxicity.

AgNPs as well as the ions released from their dissolution have tendency to adhere to the lipid membrane and generate extracellular ROS leading to its destabilization and destruction [55,56]. However, the mitochondrial toxicity and changes in its permeability remains major source of intracellular ROS. Hence, using JC-1 dye [57], we tested the mitochondrial status in untreated and V-AgNPs treated 2D cultured A549 cells (Fig. 5B). JC-1 dye has two distinct fluorescence emission spectra depending on its dissociated form (monomeric JC-1) and associated form (JC-1 aggregates). In healthy cells, owing to normal mitochondrial transmembrane potential, JC-1 enters into mitochondria and instantly forms aggregates form that show red fluorescence (λ_em_: 596 nm) [57]. On the other hand, in compromised and apoptotic cells, the mitochondrial transmembrane potential drops due to increased permeability of the mitochondrial membrane. Due to this drop, the JC-1 remains in monomeric form, showing green fluorescence (λ_em_: 520 nm) [57]. Thus, the ‘red/green fluorescence’ ratio serves as a proxy for mitochondrial polarity and by consequence, the “health” status of mitochondria [57]. For 2D cultured A549 cells, we observed high red/green ratio for untreated cells suggesting healthy mitochondria and it was reduced significantly in LD25-2D and LD50-2D treated cells (one-way ANOVA, p-value <0.05) (Fig. 5B–Supporting information, Figure S11). This implies that V-AgNPs act in cytoplasm, interact with mitochondria, and alter their permeability leading to their depolarization. This ultimately leads to energy imbalance and initiates the cascade of reactions that contribute to oxidative stress. It is possible that the silver ions released from V-AgNPs, can contribute to their interaction and toxicity in mitochondria [58,59]. But determining the extent of their contribution in nanoparticle mediated toxicity is experimentally impossible at present.

Further, we decided to test if V-AgNPs have any action on DNA of A549 cells. For the same, first we used simple Hoechst staining method [60] for 2D cultured A549 cells. As shown in Fig. 5C, the untreated cells showed blue, fluorescent intact nucleus with oblong or oval shape and seldom fragmented shape. But in LD25-2D and LD50-2D treated cells, many nuclei were shrunken, fragmented and had irregular shapes. Thus, we concluded that the V-AgNPs caused nuclear condensation and nuclear fragmentation in A549 cells. Under normal physiological conditions, the process of cell growth and division to form 2 daughter cells is represented by four intricate phases of the cell cycle: G0/G1-phase, S-phase, G2-phase, and M-phase [[61], [62], [63]]. The G0-phase represents quiescent or resting phase from which cells enter into G1-phase. With the help of several growth factors, enzymes like cyclin-dependent kinases, and intra-as well as extracellular signals, cell prepares itself for transition into S-phase. After entering the S-phase, cell synthesizes DNA for two daughter cells. In G2-phase, cell synthesizes proteins essential for mitosis process i.e. M-phase. In M-phase, cell divides forming two daughter cells which again grow and either entire G0-or G1-phase. Every cell faces two checkpoints each before entering S-phase and G2-phase. If DNA is damaged, then cell tries to repair it. Failure at any checkpoint results in initiation of apoptosis and cell death [61,64,65]. In cancer, the cell cycle is abnormal and the cancer cell escapes the checkpoint leading to abnormal and uncontrolled cell division and proliferation [[66], [67], [68]]. In any case (normal physiology or cancer), arrest is observed in a cell cycle phase owing to several reasons such as DNA damage, mitochondrial dysfunctioning, oxidative stress, abnormality in protein synthesis, genetic modifications that occur via intrinsic and/or extrinsic factors [[66], [67], [68]]. If V-AgNPs are indeed interacting and fragmenting the DNA of the cells along with mitochondrial dysfunctioning, it would be reflected in the cell cycle phases during cell division. Hence, we performed cell cycle analysis using a DNA binding dye (PI) and flow cytometry for both 2D cultured A549 cells and A549 spheroids (Fig. 5D–Supporting information, Figure S12). We observed significant decrease in G0/G1-phase population from 53% to 34% for 2D cultured A549 cells and from 87% to 74% for A549 spheroids upon V-AgNPs treatment at LD25-2D, LD50-2D, LD25-3D, and LD50-3D (one-way ANOVA, p-value <0.05). Interestingly, in 2D cultured A549 cells, in parallel to decline in G0/G1 population, we observed significant rise in S-phase population only in LD25-2D (45%) as compared to that in LD50-2D (38%). The probable reason behind this could be an attempt of the DNA synthesis machinery to cope up with DNA damage caused by LD25-2D of V-AgNPs. At LD50-2D, we observed significantly higher percentage of G2/M-phase population (26%) as compared to both untreated and LD25-2D treated cells (10% and 21%, respectively). It is proven that the DNA integrity and extent of DNA damage is scrutinized for mother cell at G2 checkpoint before allowing it to enter in mitosis phase [65,66]. With our observations, we concluded that V-AgNPs at LD50-2D caused DNA damage to such an extent that the cells were arrested in G2/M-phase while halting proliferation process. In A549 spheroids, cell cycle analysis revealed a similar trend. Concomitant to the decrease in G1 population (from 87% in untreated spheroids to 74% in treated spheroids), the S-phase population rose significantly (on-way ANOVA, p-value <0.05). It went from 11% (for untreated spheroids) to 21% (for LD25-3D) and to 25% (for LD50-3D). No changes were observed in G2/M population (one-way ANOVA, p-value >0.05) upon V-AgNPs treatment. These results suggest that the cells in spheroids were arrested at first checkpoint (G1 checkpoint) for the repairing of damaged DNA. Our flow cytometry data corroborates very well with hoechst staining data confirming DNA toxicity of our nanoparticles.

The mitochondrial dysfunctioning, DNA toxicity, and elevated ROS levels, are classical signs for the initiation of intrinsic pathway of the apoptosis process [[69], [70], [71]]. The mitochondrial membrane permeability and the transmembrane potential are tightly regulated by the balance between anti-apoptotic proteins and pro-apoptotic proteins. In normal physiology, this balance is in favor of anti-apoptotic proteins such as BCL-2 family proteins that help in survival, growth, and proliferation of cells [[69], [70], [71], [72]]. In cancer, anti-apoptotic BCL-2 family proteins are overexpressed leading to escape of cancerous cell from apoptosis and uncontrolled growth of tumor in the body [[73], [74], [75]]. Overexpression of these proteins also contributes to the resistance of tumor to most of the chemotherapeutic agents [[73], [74], [75]]. As in our previous experiments V-AgNPs showed dose-dependent cytotoxicity along with mitochondrial dysfunctioning and DNA toxicity, we decided to check the expression of two proteins: BCL-2 and BAX that are key members of anti-apoptotic proteins and pro-apoptotic proteins, respectively. Upon performing western blot analysis, we observed significant reduction in expression of BCL-2 protein (*t*-test, p-value <0.05) in cells treated with LD50-2D of V-AgNPs as compared to untreated cells (Supporting information, Figure S13). Furthermore the ratio of BAX/BCL-2 protein, which is a representative of balance of pro-apoptotic to anti-apoptotic factors was also increased significantly (*t*-test, p-value <0.05) in case of LD50-2D treated cells (Fig. 5E).

Finally, we decided to check the activation of executioner caspases i.e. caspase 3/7 enzymes in both 2D cultured A549 cells as well as A549 spheroids. Upon treatment with ReadyProbe™ Reagent, the V-AgNPs showed dose dependent expression and activation of caspase 3/7 in the form of significant increase in the green fluorescence as compared to untreated cells (Fig. 5F–Supporting information, Figure S14). Similarly, in case of A549 spheroids, LD25-3D showed elevated green fluorescence just like 2D cultured A549 cells indicating increased activation of executioner caspases (Fig. 5G). The line profile for activation of these caspases in spheroids showed that the green fluorescence intensity was higher in peripheral as well as central region of spheroid as compared to untreated suggesting action of V-AgNPs and activation of executioner caspases in entire structure of the A549 spheroid (Supporting information, Figure S15). This in turn also suggest that, our V-AgNPs are probably capable of entering into deeper central regions of the spheroids. We believe that in future, V-AgNPs could be surface decorated with targeting moiety in order to make them specific against lung cancer which will also confirm their access into central regions of the spheroids.

We also subjected the V-AgNPs treated A549 spheroids to TEM for analyzing the presence of nanoparticles and ultrastructural cellular changes upon treatment. From TEM images, we observed that V-AgNPs were presented in clustered forms in the cytoplasm and nucleus of the cell as well as in close vicinity of mitochondria (red arrows, Fig. 5H). The mitochondria had poor structural integrity and showed disrupted cristae (blue arrows, Fig. 5H). The electron micrograph also showed dead cells that have lost their cytoplasmic contents (yellow arrows, Fig. 5H). Overall, our electron microscopic data for A549 spheroids displayed almost all abnormalities in cellular morphology due to presence of V-AgNPs.

Based on the gathered evidence, we propose that V-AgNPs exert their cytotoxicity in A549 cells and A549 spheroids via the molecular axis of intrinsic apoptosis pathway. As mentioned earlier, the mitochondrial membrane permeability is critically controlled via anti-apoptotic/pro-apoptotic protein balance i.e. BCL-2/BAX ratio [[69], [70], [71], [72]]. However, DNA damage and subsequent mitochondrial distress caused by V-AgNPs lead to activation of BAX protein and BCL-2 antagonist/killer 1 (BAK) protein via several proteins such as BH3-interacting death agonist (BID), p53 upregulated modulator of apoptosis (PUMA), and BCL-2-interacting mediator (BIM) [72,75]. Under normal circumstances, the BAX protein exists in quiescent or inactive form and shuffles between outer mitochondrial membrane and cytoplasm, whereas BAK remains inactive in the outer membrane of mitochondria. But the aforementioned cellular stresses bring the conformational change in BAX and BAK which ultimately leads to their oligomerization and formation of pores in the mitochondrial membranes resulting in increased permeability [69,71,72]. The main anti-apoptotic protein BCL-2, that has capability to interact and to keep BAX/BAK proteins in their inactive forms, fails in its function due to (i) reduced expression as well as (ii) failure in its post-translational modification that renders it inactive [70]. Once, the ‘point-of-no-return’ (in this case, increased mitochondrial permeability) is attained, the cytochrome *c* starts leaking out of mitochondria into cytoplasm. This triggers the activation of caspases via either apoptotic protease activating factor-1 (APAF1) or second-mitochondria-derived activator of caspases (SMAC). APAF1 forms the apoptosome and SMAC inhibit caspase inhibitory proteins [72,76]. All this cascade eventually leads to formation of active form of executioner caspase (Caspase 3) leading to cell death [[69], [70], [71], [72],^76^].

## 4 Conclusion

Lung cancer being one of the most life-threatening diseases still has no guaranteed therapy. Thus, in an attempt to find better cancer therapeutic modalities, nanomaterials have offered tremendous advantages over conventional therapies. Among these nanomaterials, AgNPs have emerged to be an excellent choice due to their inherent cytotoxic activity. The AgNPs can be synthesized using green, environment friendly methods in large quantities with high reproducibility. Here, in the presented work, we have synthesized AgNPs from a novel green resource (Soil bacteria: *Viridibacillus* sp.). Using conventional 2D cell culture and 3D spheroidal culture of lung cancer cell line (A549 cell line), we showed that these nanoparticles (V-AgNPs) exert dose dependent cytotoxic effects. Due to the compact and tight cellular mass in A549 spheroids, the LD50 in spheroids (LD50-3D) was higher than LD50 in 2D cultured A549 cells (LD50-2D). In both 2D and 3D cell models of lung cancer, V-AgNPs specifically damaged the DNA and mitochondria leading to oxidative and replication machinery stress. V-AgNPs altered protein expression levels causing reduction in anti-apoptotic protein level. Eventually all these effects led to cell apoptosis in A549 cells and A549 spheroids via mitochondrial depolarization dependent intrinsic pathway. In future, these V-AgNPs can be further developed into a powerful highly specific nanomedicine by attaching a targeting moiety with variety of bioconjugation techniques that could make them specific while reducing off-target effects simultaneously. Furthermore, using such novel green nanoparticles along with classical anticancer drugs a synergistic combination can also be studied. Overall, these V-AgNPs offer better promises for developing silver nanomaterial based anticancer therapies.

## Funding

This work was supported by the European Union's 10.13039/501100007601Horizon 2020 research and innovation programme under the 10.13039/100010665Marie Skłodowska-Curie grant agreement No 955626 (to I.M.), the 10.13039/501100009708Novo Nordisk Foundation grant NNF20CC0035580 (to I.M.) and 10.13039/501100004785NordForsk project 105121 (to I.M.).

## CRediT authorship contribution statement

**Abhayraj S. Joshi:** Writing – review & editing, Writing – original draft, Validation, Methodology, Investigation, Formal analysis, Conceptualization. **Mugdha V. Bapat:** Writing – review & editing, Writing – original draft, Methodology, Investigation, Formal analysis. **Priyanka Singh:** Writing – review & editing, Methodology. **Ivan Mijakovic:** Writing – review & editing, Writing – original draft, Supervision, Resources, Project administration, Funding acquisition, Conceptualization.

## Declaration of competing interest

The authors declare that they have no known competing financial interests or personal relationships that could have appeared to influence the work reported in this paper.

## Data Availability

Data will be made available on request.
