# Supplementary material for: Viridibacillus culture derived silver nanoparticles exert potent anticancer action in 2D and 3D models of lung cancer via mitochondrial depolarization-mediated apoptosis
Source: Mater Today Bio. 2024 Feb 11;25:100997. doi: 10.1016/j.mtbio.2024.100997 (PMC10876681; doi:10.1016/j.mtbio.2024.100997)
Supplement: Multimedia component 1 [file mmc1.docx]

**Supplementary Information**

***Viridibacillus* culture derived silver nanoparticles exert potent anticancer action in 2D and 3D models of lung cancer via mitochondrial depolarization-mediated apoptosis**

Abhayraj S. Joshi**^† a^**, Mugdha V. Bapat**^†a^**, Priyanka Singh**^a^**, and Ivan Mijakovic*** ^a, b^**

**^a^** The Novo Nordisk Foundation Center for Biosustainability, Technical University of Denmark, Kongens Lyngby, Denmark; [abshjo@biosustain.dtu.dk](mailto:abshjo@biosustain.dtu.dk), [bapat.mugdha26@gmail.com,](mailto:bapat.mugdha26@gmail.com,) prisin@biosustain.dtu.dk

**^b^** Department of Biology and Biological Engineering, Division of Systems and Synthetic Biology, Chalmers University of Technology, Sweden; [ivan.mijakovic@chalmers.se](mailto:ivan.mijakovic@chalmers.se)

**^†^** Co-first authors who contributing equally.

***** Correspondence: [ivan.mijakovic@chalmers.se](mailto:ivan.mijakovic@chalmers.se)


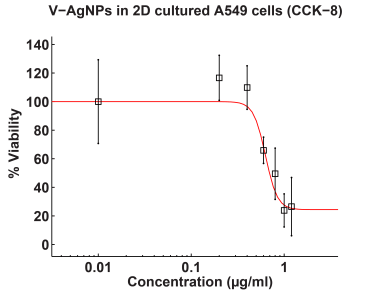


**Figure S1: Curve fitting performed using Matlab-GUI based Dr. Fit program for analysis of cell viability data obtained in 2D cultured A549 cells using CCK-8 cell viability kit to determine LD50-2D concentration accurately.**


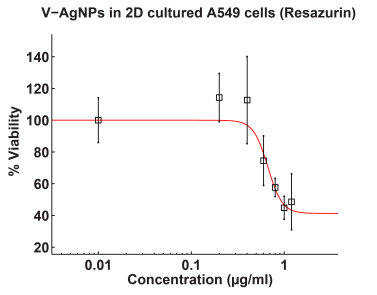


**Figure S2: Curve fitting performed using Matlab-GUI based Dr. Fit program for analysis of cell viability data obtained in 2D cultured A549 cells using resazurin dye to determine LD50-2D concentration accurately.**


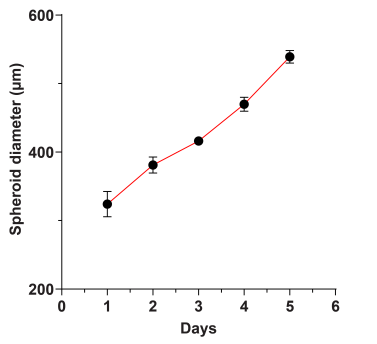


**Figure S3: Growth kinetics of A549 spheroids obtained from daily measurement of the spheroid diameter (µm).**


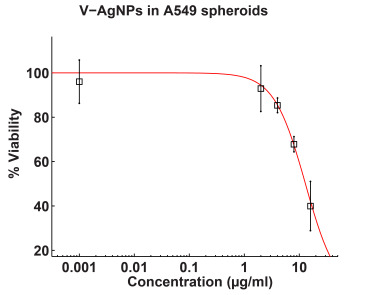


**Figure S4: Curve fitting performed using Matlab-GUI based Dr. Fit program for analysis of cell viability data obtained in 3D cultured A549 cells (A549 spheroids) using Cell Titer Glo® 3D viability kit to determine LD50-3D concentration accurately.**


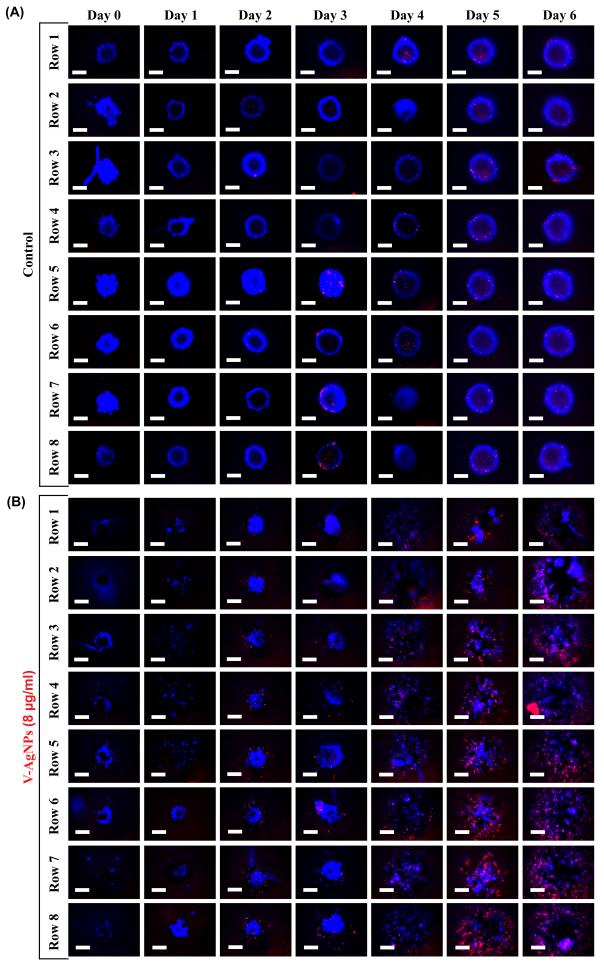


**Figure S5: *In vitro* tumor progression model showing the growth of spheroids in absence and presence of V-AgNPs.**

The growth of total 8 spheroids was monitored every day and those spheroids were imaged after staining with hoechst (blue fluorescence) and ethidium homodimer II (red fluorescence) for determining the cell death. Representative images of 3 spheroids per group have been given in figure 3B of the manuscript; whereas the kinetics of this data are represented in figure 3C (black line for control and red line for V-AgNPs treated spheroids). (Scale bar in the images: 200 µm)


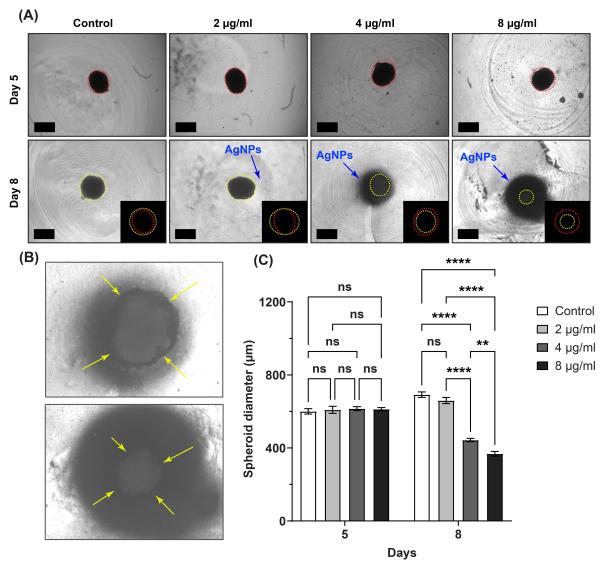


**Figure S6: *In vitro* tumor size model showing changes in the diameter of spheroids before and after the treatment with V-AgNPs at various concentrations (2-8 µg/ml).**

The 5 days old spheroids were imaged just before the treatment (day 5 row in S6-A panel) and 3 days after the treatment (day 8 row in S6-A). The insets in the image of 8^th^ day row of S6-A show qualitative differences in diameter of spheroids (red dotted line: diameter before the treatment, and yellow dotted line: diameter after the treatment). S6-B panel shows representative zoomed-in images of 4 µg/ml and 8 µg/ml V-AgNPs treated spheroids. The changes in the diameters of spheroids before and after the treatment that were measured using FIJI software from their brightfield images and analyzed using two way ANOVA test. The intra-day comparison (S6-C panel) and inter-day comparison (Figure 3F in the manuscript) were represented as bar graph showing mean ± std. error of 24 spheroids (*n*=24). (Scale bar in the images: 500 µm).


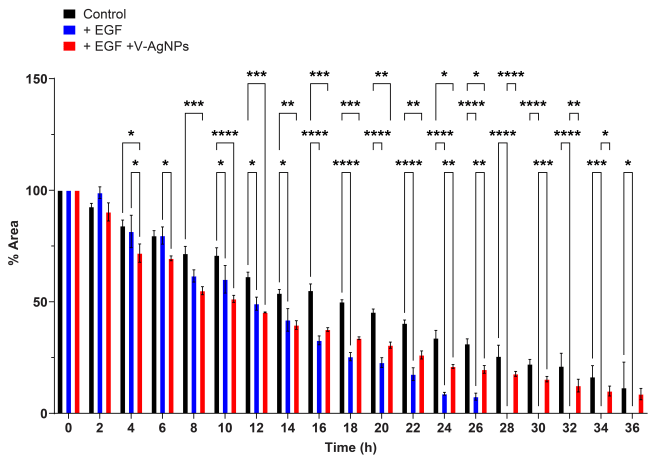


**Figure S7: The kinetics of cell migration showing time window of 4-34 h within which the cell migration differed significantly (two-way ANOVA, p-value < 0.05) from each other within untreated, EGF treated, ad EGF+V-AgNPs treated cells.**


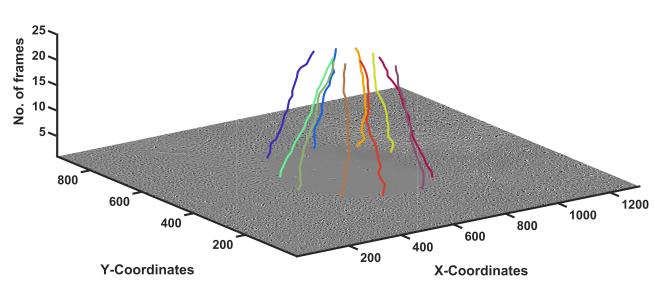


**Figure S8: 3D graph plotted using number of time frames, X-coordinates, and Y-coordinates of migrating A549 cells of control (untreated) group that were obtained from Matlab-GUI based CellTracker program to determine the directionality and velocity of migrating cells.**


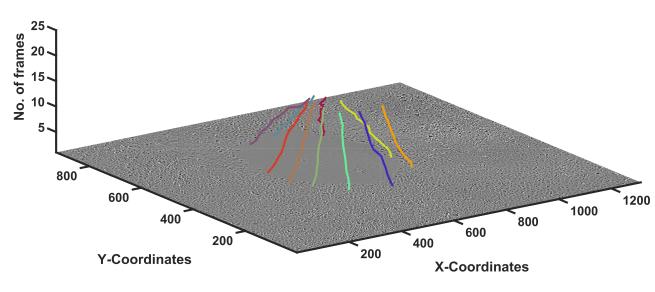


**Figure S9: 3D graph plotted using number of time frames, X-coordinates, and Y-coordinates of migrating A549 cells of EGF treated group that were obtained from Matlab-GUI based CellTracker program to determine the directionality and velocity of migrating cells.**


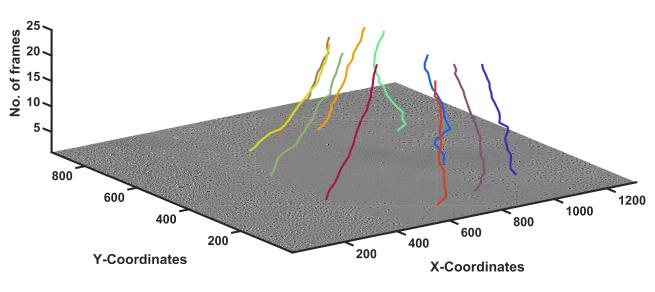


**Figure S10: 3D graph plotted using number of time frames, X-coordinates, and Y-coordinates of migrating A549 cells of EGF and V-AgNPs treated group that were obtained from Matlab-GUI based CellTracker program to determine the directionality and velocity of migrating cells.**


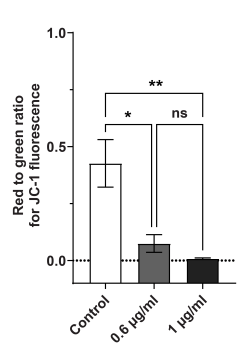


**Figure S11: Quantification of red and green fluorescence intensity for JC-1 dye in untreated (control) and V-AgNPs treated (LD25-2D and LD50-2D) A549 cells.**

The representative images are given in figure 5B of the manuscript. The bar graph here represents mean ± std. error of 4 replicates (*n*=4).


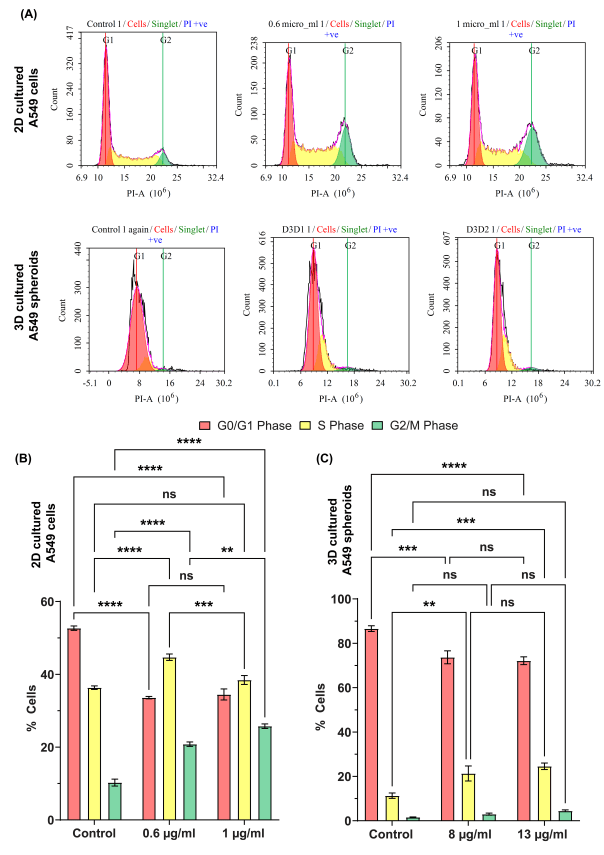


**Figure S12: Cell cycle analysis of 2D cultured A549 cells and 3D A549 spheroids by flow cytometry.**

The S12-A panel shows representative histograms and cell cycle fitting for control, LD25-2D, and LD50-2D treated A549 cells (upper row) as well as for control, LD25-3D, and LD50-3D treated A549 spheroids (lower row). The percentage of cells in each phase were calculated from Dean-Jett-Fox (DJF) algorithm and their means ± std. errors were represented as bar graphs. The data obtained from 3 replicates (*n*=3) were analyzed using two-way ANOVA (S12-B and S12-C panels). The percentage of cells arrested in each phase of cell cycle were represented in the form of pie chart in figure 5D of the manuscript.


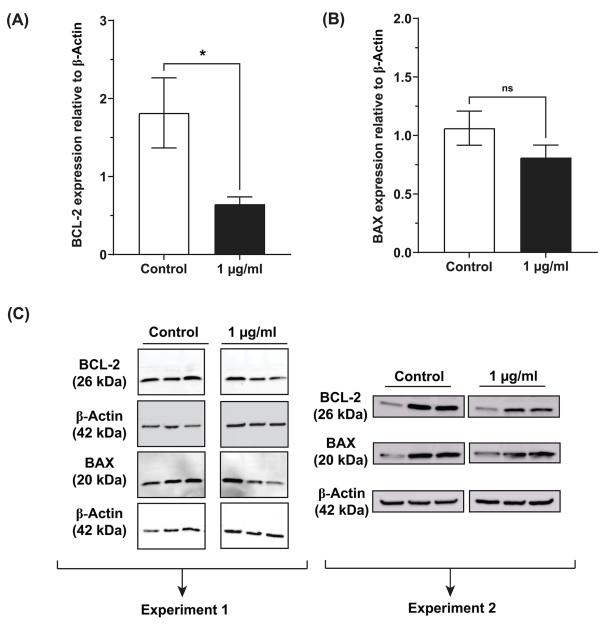


**Figure S13: Quantification of protein expression from western blot of 2D cultured A549 cells relative to β-actin.**

S13-A and S13-B show BCL-2 and BAX expression in untreated and LD50-2D treated A549 cells, respectively. S13-C shows the western blot images of two experiments, each conducted with three biological replicates. Here, the bars in each graph represent mean ± std. error of 6 replicates (*n*=6). The gel images and the bar graph of BAX/BCL-2 expression ratio is given in figure 5E and 5H of the main manuscript, respectively.


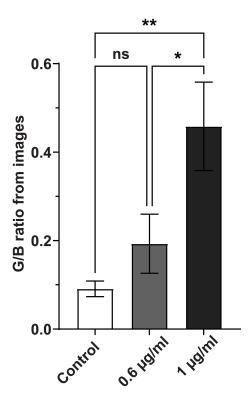


**Figure S14: Quantification of caspase 3/7 activation in untreated and V-AgNPs treated 2D cultured A549 cells.**

The bar graph represents ratio of green fluorescence (denoting caspase 3/7 activation) and blue fluorescence (denoting DAPI stained nuclei). The green and blue fluorescence intensities were calculated from 6 images per group and the data are represented as mean ± std. error of 6 replicates (*n*=6). The representative cell images are given in figure 5F of the manuscript.


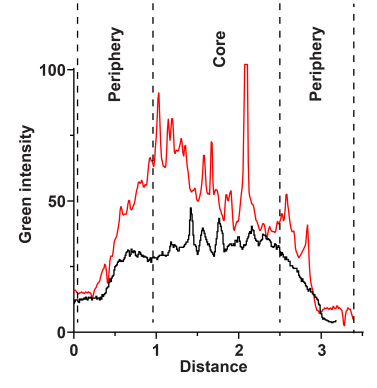


**Figure S15: The line profile for green fluorescence intensity that denotes caspase 3/7 activation in untreated (black) and V-AgNPs treated (red) A549 spheroids.**

The line graph represents mean green intensity of 3 replicates (*n*=3). V-AgNPs treatment leads to activation of executioner caspases (caspase 3/7) in the peripheral as well as central regions of spheroids unlike control untreated spheroids confirming cytocidal activity of V-AgNPs in 3D models of lung cancer i.e. A549 spheroids. The representative images of untreated and V-AgNPs treated spheroids are given in figure 5G of the manuscript.
